# Supplementary material for: Changes in medical student attendance and its impact on student educational outcomes: a systematic review protocol
Source: BMJ Open. 2024 Mar 11;14(3):e078252. doi: 10.1136/bmjopen-2023-078252 (PMC10936488; doi:10.1136/bmjopen-2023-078252)
Supplement: Supplementary data [file bmjopen-2023-078252supp002.pdf]

Supplementary Material 2

Search Strategies

Medline via Ovid

Ovid MEDLINE(R) and Epub Ahead of Print, In-Process, In-Data-Review & Other Non-Indexed Citations, Daily and Versions <1946 to September 20, 2023>		
1	((clinical or medical or doctor) adj2 (student* or trainee* or school*)).ti,ab.	90738
2	exp Schools, Medical/ or exp Students, Medical/ or exp education, medical, undergraduate/	80136
3	1 or 2	126117
4	(Absen* or non-attendance or nonattendance or attend*).ti,ab.	1079900
5	(Placemen* or Lectur* or Semina* or online or virtual or teaching or "in person" or inperson or "face to face" or traditional or class*).ti,ab.	2761182
6	exp Teaching/	93309
7	5 or 6	2811415
8	(Exam* or Perform* or Attainment* or Result*).ti,ab.	15523220
9	exp academic performance/ or exp educational status/ or exp academic success/	65300
10	8 or 9	15540736
11	3 and 4 and 7 and 10	3160

Embase via Ovid

Embase <1974 to 2023 September 20>		
1	((clinical or medical or doctor) adj2 (student* or trainee* or school*)).ti,ab. or exp *medical school/ or exp *medical student/	131819
2	(Absen* or non-attendance or nonattendance or attend*).ti,ab. or exp *school attendance/	1459034
3	(Placemen* or Lectur* or Semina* or online or virtual or teaching or "in person" or inperson or "face to face" or traditional or class*).ti,ab. or exp *Teaching/	3657834
4	exp academic achievement/ or exp *educational status/ or (Exam* or Perform* or Attainment* or Result*).ti,ab.	20509010
5	1 and 2 and 3 and 4	5027
6	limit 5 to embase	1697

Web of Science

```
# Web of Science Search Strategy (v0.1)
# Database: Web of Science Core Collection
# Entitlements:
- WOS.IC: 1993 to 2023
- WOS.CCR: 1985 to 2023
- WOS.SCI: 1900 to 2023
- WOS.AHCI: 1975 to 2023
- WOS.BHCI: 2008 to 2023
- WOS.BSCI: 2008 to 2023
```

- WOS.ESCI: 2018 to 2023<br>- WOS.ISTP: 1990 to 2023<br>- WOS.SSCI: 1956 to 2023<br>- WOS.ISSHP: 1990 to 2023<br>Date Run: Thu Sep 20, 2023		
1	TS=(Absen* OR "non-attendance" OR attendance)	1135903
2	TS= ((clinical OR medical OR doctor) near/2 (student* OR trainee* OR school*))	97987
3	TS=(Placemen* OR Lectur* OR Semina* OR online OR virtual OR teaching or "in person" or inperson or "face to face" or traditional or class*)	6854033
4	TS=(Exam* OR Perform* OR Attainment* OR Result* OR "academic performance" OR "educational status")	6854033
5	TS=(Exam* OR Perform* OR Attainment* OR Result* OR "academic performance" OR "educational status")	29046391
6	#4 AND #3 AND #2 AND #1	811

Scopus

Scopus<br>Date Run: Thu Sep 20, 2023	
1	TITLE-ABS-KEY (((absen* OR "non-attend*" OR attend* OR nonattend*) AND ((clinical OR medical OR doctor) W/2 (student* OR trainee* OR school*)) AND (placemen* OR lectur* OR semina* OR online OR virtual OR teaching OR "in person" OR inperson OR "face to face" OR traditional OR class*) AND (exam* OR perform* OR attainment* OR result* OR "academic performance" OR "educational status")))

British Education Index via EBSCOhost

Interface - EBSCOhost Research Databases<br>Search Screen - Advanced<br>Search Database - British Education Index<br><br>Expanders - Apply equivalent subjects<br>Search modes - Boolean/Phrase<br>Date Run: Thu Sep 20, 2023		
S1	TI ((clinical or medical or doctor) w2 (student* or trainee* or school*)) OR AB ((clinical or medical or doctor) w2 (student* or trainee* or school*))	4,076
S2	((DE "MEDICAL schools") OR (DE "MEDICAL students") OR (DE "MEDICAL education"))	9,405
S3	S1 OR S2	10,242
S4	TI ((Absen* or non-attendance or nonattendance or attend*)) OR ((Absen* or non-attendance or nonattendance or attend*))	7,649

S5	DE "SCHOOL attendance"	610
S6	S4 OR S5	7,649
S7	TI ((Placemen* or Lectur* or Semina* or online or virtual or teaching or "in person" or inperson or "face to face" or traditional or class*)) OR AB ((Placemen* or Lectur* or Semina* or online or virtual or teaching or "in person" or inperson or "face to face" or traditional or class*))	76,866
S8	DE "TEACHING"	6,709
S9	S7 OR S8	79,470
S10	TI ((Exam* or Perform* or Attainment* or Result*)) OR AB ((Exam* or Perform* or Attainment* or Result*))	80,327
S11	(DE "ACADEMIC achievement") OR (DE "EDUCATIONAL attainment")	10,645
S12	S10 OR S11	85,240
S13	S3 AND S6 AND S9 AND S12	101

ERIC via EBSCOhost

Interface - EBSCOhost Research Databases<br>Search Screen - Advanced<br>Search Database - ERIC<br><br>Expanders - Apply equivalent subjects<br>Search modes - Boolean/Phrase<br>Date Run: Thu Sep 20, 2023		
S1	(AB (clinical or medical or doctor) w2 (student* or trainee* or school*)) OR (TI (clinical or medical or doctor) w2 (student* or trainee* or school*))	7,784
S2	TI (DE "Medical Schools") OR (DE "Medical Students") OR (DE "Medical Education")	397
S3	S1 OR S2	7,842
S4	(AB (Absen* or non-attendance or nonattendance or attend*)) OR (TI (Absen* or non-attendance or nonattendance or attend*))	73,324
S5	(AB (Placemen* or Lectur* or Semina* or online or virtual or teaching or "in person" or inperson or "face to face" or traditional or class*)) OR (TI (Placemen* or Lectur*	672,070

	or Semina* or online or virtual or teaching or "in person" or inperson or "face to face" or traditional or class*))	
S6	DE "Teaching Methods"	211,764
S7	S5 OR S6	737,112
S8	(AB (Exam* or Perform* or Attainment* or Result*)) OR (TI (Exam* or Perform* or Attainment* or Result*))	845,630
S9	DE "Academic Achievement"	94,882
S10	S8 OR S9	876,868
S11	S3 AND S4 AND S7 AND S10	138
