# Supplementary material for: Changes in medical student attendance and its impact on student educational outcomes: a systematic review protocol
Source: BMJ Open. 2024 Mar 11;14(3):e078252. doi: 10.1136/bmjopen-2023-078252 (PMC10936488; doi:10.1136/bmjopen-2023-078252)
Supplement: Author's manuscript [file bmjopen-2023-078252.draft_revisions.pdf]

BMJ Open

BMJ Open is committed to open peer review. As part of this commitment we make the peer review history of every article we publish publicly available.

When an article is published we post the peer reviewers' comments and the authors' responses online. We also post the versions of the paper that were used during peer review. These are the versions that the peer review comments apply to.

The versions of the paper that follow are the versions that were submitted during the peer review process. They are not the versions of record or the final published versions. They should not be cited or distributed as the published version of this manuscript.

BMJ Open is an open access journal and the full, final, typeset and author-corrected version of record of the manuscript is available on our site with no access controls, subscription charges or pay-per-view fees (http://bmjopen.bmj.com).

If you have any questions on BMJ Open's open peer review process please email info.bmjopen@bmj.com

BMJ Open

Medical school teaching attendance in an environment of increased online learning.

Journal:	*BMJ Open*
Manuscript ID	bmjopen-2023-078252
Article Type:	Protocol
Date Submitted by the Author:	28-Jul-2023
Complete List of Authors:	Nagappan, Palaniappan Ganesh; University of Cambridge, School of Clinical Medicine<br>Brown, Samuel; University of Cambridge<br>McManus, Alex; University of Cambridge<br>Sayers, Sarah; University of Cambridge,<br>Absar, Shazia; University of Cambridge<br>Tan, Sapphire; University of Cambridge; University of Cambridge<br>Tulinius, Charlotte; University of Cambridge<br>Lau, Edward; University of Cambridge
Keywords:	EDUCATION & TRAINING (see Medical Education & Training), Health Education, MEDICAL EDUCATION & TRAINING

SCHOLARONE™
Manuscripts

Attendance Review Protocol

Contents

Administrative information 2
- Title 2
- Authors 2
- Support 2

Introduction 2
- Rationale 2
- Objectives 3

Methods 3
- Eligibility criteria 3
- Information sources 3
- Search strategy 4
- Study records 5
  - Data management 5
  - Selection process 5
  - Data collection process 5
- Data items 6
- Outcomes and prioritization 6
- Risk of bias in individual studies 7
- Data synthesis 7
- Meta-bias(es) 8
- Confidence in cumulative evidence 8

For peer review only

This template was created by Emily Jones (epjones3@email.unc.edu). It can be used non-commercially [illegible]l and research purposes if attribution is provided. Updated 6/22/2022.

Administrative information

Title

Medical school teaching attendance in an environment of increased online learning.

Authors

Dr Palaniappan Ganesh Nagappan. University of Cambridge:

pgn25@cam.ac.uk

Mr Samuel Brown. University of Cambridge: sb2382@cam.ac.uk

Mr Alex McManus. University of Cambridge: am2776@cam.ac.uk

Miss Sarah Sayers. University of Cambridge: sbs51@cam.ac.uk

Miss Shazia Absar. University of Cambridge: sha36@cam.ac.uk

Dr Sapphire Tan Rou Xi, University of Cambridge: rxst2@cam.ac.uk

Miss Isla Kuhn. University of Cambridge: ilk21@cam.ac.uk

Dr Charlotte Tulinius. University of Cambridge: ct419@medschl.cam.ac.uk

Dr Edward Lau. University of Cambridge: ejsl2@cam.ac.uk

Corresponding author:

Dr Palaniappan Ganesh Nagappan
Emmanuel College
Cambridge, CB23AP, UK
+447472659583
Pgn25@cam.ac.uk

Word Count: 1992 words

Abstract

Introduction

Post-pandemic, medical student attendance at Cambridge University and other UK medical schools has dramatically decreased, with a similar trend observed globally. The shift to online learning during the pandemic has led some students to prefer remote education over in-person teaching, despite the challenges of online learning. Our research aims to understand the reasons behind this shift and its impact on students' performance to find potential solutions for improving learning outcomes.

For peer review only

Methods and analysis

The systematic review will follow the guidelines of the Centre of Research and Dissemination, Moose, and PRISMA. The study aims to investigate changes in attendance and performance among medical students in the context of increased online learning. Eligibility criteria include medical students from various universities, both graduate and undergraduate, with recorded evidence of attendance changes and comparison of performance measures. Data will be managed using Endnote and NVIVO, and two pairs of reviewers will independently perform screening and data extraction, with a third reviewer for dispute resolution. Risk of bias in individual studies will be assessed using the Newcastle-Ottawa Scale, and data synthesis will be conducted through qualitative and narrative approaches. Confidence in cumulative evidence will be evaluated using the GRADE-CERQual approach.

Ethics and dissemination

The collated and analysed data will be published and subsequently be used to guide focused group discussions.

Article Summary

Strengths and limitations of this study

- The study's adherence to comprehensive methodology, following established guidelines like Centre of Research and Dissemination, Moose, and PRISMA, ensures a systematic and rigorous approach, enhancing credibility and reliability.
- The inclusion of diverse research types (qualitative, quantitative, mixed methods) provides a comprehensive understanding, allowing triangulation of evidence and strengthening the validity of conclusions.
- Independent data management and extraction by two pairs of reviewers reduce biases and enhance objectivity, leading to more robust and credible research outcomes.
- The presence of limited literature on the specific topic may impact the depth of findings and necessitates careful consideration of implications.
- Potential language bias and exclusion of grey literature could lead to oversight of relevant studies, and addressing these limitations is essential for a more comprehensive analysis.

Keywords

- Online Learning (MeSH Term: Education, Distance)
- Medical Students (MeSH Term: Students, Medical)
- Attendance (MeSH Term: Absenteeism)
- Performance (MeSH Term: Academic Performance)

For peer review only

Introduction

Rationale

Post-pandemic, the level of Y4-Y6 and CGC medical students' attendance at Cambridge University seem to have fallen dramatically. University of Cambridge School of Clinical Medicine teachers and leads across all subjects report a very low attendance rate for teaching in small groups, larger groups as well as for whole cohort lectures. There have been reports of similar phenomena across other medical schools in the UK. The literature is still limited on this development, but this is expected to be a worldwide development.

Pre-pandemic, most students would have expected to be 'at school/in clinical placements' all day, but the pandemic introduced the need for replacement of in-person education, creative experiments with learning online and from home, and application of telemedicine (1). Some of the current medical students have only experienced higher education teaching and learning online and often asynchronously delivered. During the pandemic, the downfalls of online teaching were seen as devastating for both students and teachers (2), and it seems almost paradoxical that students are now actively opting out of in-person teaching.

A US study (3) concludes that the post-pandemic decline in attendance is caused by students being more inclined to take over control of where and when they want to learn, but also calls for more research to explain the phenomenon.

Objectives

To synthesise the existing evidence available on why attendance is changing in medical schools in an environment of increased online learning and its relation to their performance as medical students. The secondary objective is to find solutions to increase performance and learning.

Completed	PRISMA-P Checklist Item	Description
	7	Provide an explicit statement of the question(s) the review will address with reference to participants, interventions, comparators, and outcomes (PICO)

Methods

The design and methods used for this systematic review comply with Centre of Research and Dissemination Guidelines, Meta-analyses of Observational Studies in Epidemiology (Moose) and are reported in line with Preferred Reporting Items for Systematic Reviews and Meta-Analyses (PRISMA). Eligibility criteria were informed using the SPIDER and MOOSE guidelines.

Eligibility criteria

(S) Sample: Medical students in both graduate and undergraduate medical curriculums across all types of universities (private and public)

For peer review only - http://bmjopen.bmj.com/site/about/guidelines.xhtml

(PI) The phenomenon of Interest: changes in attendance, performance, or both in an environment of increased online learning.

(E) Evaluation: 1. Recorded or anecdotal evidence of changes in attendance and 2. Comparison of examination scores or any other performance-based measures.

(D) Design: All types of studies excluding grey literature.

(R) Research type: qualitative, quantitative and mixed-methods research could be searched for.

Completed	PRISMA-P Checklist Item	Description
	8	Specify the study characteristics (such as PICO, study design, setting, time frame) and report characteristics (such as years considered, language, publication status) to be used as criteria for eligibility for the review

Information sources

The search will employ topic-based strategies designed for each database from inception to 1st February 2023. There will be no language or geographical restrictions.

Databases:

- EMBASE via OVID, MEDLINE via OVID, Scopus, Web of Science Core Collection, British Education Index via EBSCOhost, ERIC via EBSCOhost

Completed	PRISMA-P Checklist Item	Description
	9	Describe all intended information sources (such as electronic databases, contact with study authors, trial registers or other grey literature sources) with planned dates of coverage

Search strategy

Medline via Ovid

(((clinical or medical or doctor) adj2 (student* or trainee* or school*)).ti,ab. or exp Schools, Medical/ or exp Students, Medical/ or exp education, medical, undergraduate/) and (Absen* or non-attendance or nonattendance or attend*).ti,ab. and ((Placemen* or Lectur* or Semina* or online or virtual or teaching).ti,ab. or exp Teaching/) and (exp academic performance/ or exp educational status/ or exp academic success/ or (Exam* or Perform* or Attainment* or Result*).ti,ab.) 2583

Embase via Ovid

For peer review only - http://bmjopen.bmj.com/site/about/guidelines.xhtml

(((clinical or medical or doctor) adj2 (student* or trainee* or school*)).ti,ab. or exp *medical school/ or exp *medical student/) and ((Absen* or non-attendance or nonattendance or attend*).ti,ab. or exp *school attendance/) and ((Placemen* or Lectur* or Semina* or online or virtual or teaching).ti,ab. or exp *Teaching/) and (exp academic achievement/ or exp *educational status/ or (Exam* or Perform* or Attainment* or Result*).ti,ab.) 4040

Web of Science

TS=((Absen* OR "non-attendance" OR attendance) AND ((clinical OR medical OR doctor) near/2 (student* OR trainee* OR school*)) AND (Placemen* OR Lectur* OR Semina* OR online OR virtual OR teaching) AND (Exam* OR Perform* OR Attainment* OR Result* OR "academic performance" OR "educational status")) 605

Scopus

TITLE-ABS-KEY (((absen* OR "non-attend*" OR attend* OR nonattend*) AND ((clinical OR medical OR doctor) W/2 (student* OR trainee* OR school*)) AND (placemen* OR lectur* OR semina* OR online OR virtual OR teaching) AND (exam* OR perform* OR attainment* OR result* OR "academic performance" OR "educational status"))) 3243

British Education Index via EBSCOhost

#	Query - BEI	Results
S13	(S3 AND S6 AND S9 AND S12)	76
S12	(S10 OR S11)	75,049
S11	AB (Exam* or Perform* or Attainment* or Result*)	69,031
S10	(DE "ACADEMIC achievement") OR (DE "EDUCATIONAL attainment")	10,236
S9	(S7 OR S8)	37,721
S8	AB (Placemen* or Lectur* or Semina* or online or virtual or teaching)	33,800
S7	DE "TEACHING"	6,508
S6	(S4 OR S5)	6,293
S5	AB (Absen* or non-attendance or nonattendance or attend*)	5,888
S4	DE "SCHOOL attendance"	600
S3	(S1 OR S2)	9,827

For peer review only - http://bmjopen.bmj.com/site/about/guidelines.xhtml

S2	((DE "MEDICAL schools") OR (DE "MEDICAL students") OR (DE "MEDICAL education"))	9,182
S1	AB (clinical or medical or doctor) w2 (student* or trainee* or school*)	2,738

ERIC via EBSCOhost

#	Query - ERIC	Results
S13	(S3 AND S8 AND S9 AND S12)	159
S12	S10 OR S11	854,946
S11	DE "Academic Achievement"	93,901
S10	AB (Exam* or Perform* or Attainment* or Result*)	822,586
S9	S6 OR S7	468,045
S8	S4 OR S5	72,717
S7	DE "Teaching Methods"	209,699
S6	AB (Placemen* or Lectur* or Semina* or online or virtual or teaching)	355,969
S5	DE "Attendance"	5,866
S4	AB (Absen* or non-attendance or nonattendance or attend*)	71,383
S3	S1 OR S2	14,990
S2	(DE "Medical Schools") OR (DE "Medical Students") OR (DE "Medical Education")	13,358
S1	AB (clinical or medical or doctor) w2 (student* or trainee* or school*)	7,017

Completed	PRISMA-P Checklist Item	Description
	10	Present draft of search strategy to be used for at least one electronic database, including planned limits, such that it could be repeated

Study records

Data management

Records will be managed through Endnote and NVIVO.

Selection process

Inclusion criteria:

- Medical students studying in medical schools accredited by their country's governing body.
- Language of Publication: all languages
- Settings: Hospitals, medical schools
- At least one of the following outcomes: 1. attendance rates, 2. performance measures, and 3. solutions.

For peer review only - http://bmjopen.bmj.com/site/about/guidelines.xhtml

Exclusion criteria:

- Grey literature

Screening and data extraction will be performed independently by two pairs of reviewers (SS/SA; SB/AM, with each pair covering 50% of the papers, with a third individual to resolve any disagreements.

Data collection process

Using a standardised form, two pairs of reviewers (SS/SA; SB/AM) will extract the data independently, with each pair covering 50% of the papers. A third reviewer (PN) will independently check the data for consistency and clarity.

Completed	PRISMA-P Checklist Item	Description
	11a	Describe the mechanism(s) that will be used to manage records and data throughout the review
	11b	State the process that will be used for selecting studies (such as two independent reviewers) through each phase of the review (that is, screening, eligibility and inclusion in meta-analysis)
	11c	Describe planned method of extracting data from reports (such as piloting forms, done independently, in duplicate), any processes for obtaining and confirming data from investigators

Data items

Data extracted will include the following summary data:

- Sample characteristics
- Sample size
- Study date
- Medium of lecture / programme / teaching
- Stage of medical education training
- Academic outcome / performance scores
- Academic outcome / performance measure used
- Attendance rates
- Access rates to online materials
- Solutions / interventions used
- Association between attendance rates and academic outcomes / performance
- Students' perception
- Other outcomes

For peer review only - http://bmjopen.bmj.com/site/about/guidelines.xhtml

This will be in addition to the critical arguments raised during the process. Quantitative data will be collected to undergo narrative synthesis.

Completed	PRISMA-P Checklist Item	Description
	12	List and define all variables for which data will be sought (such as PICO items, funding sources), any pre-planned data assumptions and simplifications

Outcomes and prioritization

1. Recorded or anecdotal evidence of changes in attendance and

2. Comparison of examination scores or any other performance-based measures.

Completed	PRISMA-P Checklist Item	Description
	13	List and define all outcomes for which data will be sought, including prioritization of main and additional outcomes, with rationale

Risk of bias in individual studies

The risk of bias for each included trial will be independently assessed by the same initial reviewers. The fifth reviewer will mediate in situations of disagreement. Cohen's κ will be used to assess agreement between reviewers. All tools and processes will be piloted before use. The risk of bias will be assessed using the Newcastle-Ottawa Scale (4,5).

The following assessments will then be made, depending on the study type:

Qualitative studies: The reviewers will assess the appropriateness of data sources and analytical processes, the study's consideration of context/setting, and the study's consideration of researchers' influence.

Descriptive cross-sectional quantitative studies: The reviewers will assess the appropriateness of the study's sampling strategy and the representativeness of the sample, the use of appropriate measurement instruments, and the acceptability of the response rate.

Nonrandomised quantitative studies: The reviewers will assess the study's minimisation of selection bias, use of appropriate measurement instruments, use of comparable groups across study conditions, and the completeness of outcome data.

Completed	PRISMA-P Checklist Item	Description
	14	Describe anticipated methods for assessing risk of bias of individual studies, including whether this will be done at the outcome or study level, or both; state how this information will be used in data synthesis

For peer review only - http://bmjopen.bmj.com/site/about/guidelines.xhtml

For peer review only

Data synthesis

Qualitative Data: The concepts are constantly compared with the data to identify the relationships among them. NVIVO will be used to do this. The aim is to develop a synthesising argument in the form of a coherent theoretical framework including a network of constructs and the relationships between them. (6–8)

Quantitative Data: The data collected will undergo narrative synthesis. A meta-analysis will be considered should the quantitative data be sufficiently homogenous.

Completed	PRISMA-P Checklist Item	Description
	15a	Describe criteria under which study data will be quantitatively synthesised
	15b	If data are appropriate for quantitative synthesis, describe planned summary measures, methods of handling data and methods of combining data from studies, including any planned exploration of consistency (such as I^2, Kendall's τ)
	15c	Describe any proposed additional analyses (such as sensitivity or subgroup analyses, meta-regression)
	15d	If quantitative synthesis is not appropriate, describe the type of summary planned

Meta-bias(es)

N/A

Completed	PRISMA-P Checklist Item	Description
	16	Specify any planned assessment of meta-bias(es) (such as publication bias across studies, selective reporting within studies)

Confidence in cumulative evidence

Confidence in discrete review findings will be assessed using the recently developed Grading of Recommendations, Assessment, Development and Evaluation - Confidence in the Evidence from Reviews of Qualitative Research (GRADE-CERQual) approach by Lewin et al. Assessment of confidence in a given review finding involves evaluating how likely it is that the finding represents a real phenomenon, i.e. factors leading to changes in attendance, performance and their relationship with each other. This assessment will be based upon an evaluation of the following: (i) methodological limitations of the primary studies contributing to the finding, (ii) the relevance of the primary contributing studies regarding the objectives of the systematic review, (iii) the coherence of the finding, and (iv) the adequacy of data supporting the finding.

For peer review only - http://bmjopen.bmj.com/site/about/guidelines.xhtml

A summary table will list for each review finding—primary contributing studies, evaluations of the above four domains, an overall confidence rating (high, moderate, low, or very low), and an explanation of the rating judgement.

Completed	PRISMA-P Checklist Item	Description
	17	Describe how the strength of the body of evidence will be assessed (such as GRADE)

Ethics and dissemination

The collated and analysed data will be published and subsequently be used to guide focused group discussions.

Author Contributions

PGN: Drafted the proposal, conceptualisation of topic.

SB: Data collection and refining of topic.

AM: Data collection and refining of topic.

SS: Data collection and refining of topic.

SA: Data collection and refining of topic.

ST: Conceptualisation of topic.

IK: Review and critiquing to improve research methodology with the drafting of the search strategy.

CT: Conceptualisation of topic and supervision.

EL: Conceptualisation of topic and supervision.

Funding

This research received no specific grant from any funding agency in the public, commercial or not-for-profit sectors. However, the necessary Qualtrics and Nvivo licenses will be available from the University of Cambridge.

Competing interests' statement

There are no known competing interests.

For peer review only

References

1. Park H, Shim S, Lee YM. A scoping review on adaptations of clinical education for medical students during COVID-19. Prim Care Diabetes. 2021 Dec;15(6):958–76.

2. Jhajj S, Kaur P, Jhajj P, Ramadan A, Jain P, Upadhyay S, et al. Impact of Covid-19 on Medical Students around the Globe. J Community Hosp Intern Med Perspect. 2022;12(4):1–6.

3. Gardner G, Feldman M, Santen SA, Mui P, Biskobing D. Determinants and Outcomes of In-person Lecture Attendance in Medical School. Med Sci Educ. 2022 Aug;32(4):883–90.

4. Deeks JJ, Dinnes J, D'Amico R, Sowden AJ, Sakarovitch C, Song F, et al. Evaluating non-randomised intervention studies. Health Technol Assess Winch Engl. 2003;7(27):iii–173.

5. Downs SH, Black N. The feasibility of creating a checklist for the assessment of the methodological quality both of randomised and non-randomised studies of health care interventions. J Epidemiol Community Health. 1998 Jun;52(6):377–84.

6. Dixon-Woods M, Cavers D, Agarwal S, Annandale E, Arthur A, Harvey J, et al. Conducting a critical interpretive synthesis of the literature on access to healthcare by vulnerable groups. BMC Med Res Methodol. 2006 Jul 26;6(1):35.

7. Entwistle V, Firnigl D, Ryan M, Francis J, Kinghorn P. Which experiences of health care delivery matter to service users and why? A critical interpretive synthesis and conceptual map. J Health Serv Res Policy. 2012 Apr;17(2):70–8.

8. Schick-Makaroff K, MacDonald M, Plummer M, Burgess J, Neander W. What Synthesis Methodology Should I Use? A Review and Analysis of Approaches to Research Synthesis. AIMS Public Health. 2016;3(1):172–215.

For peer review only

For peer review only - http://bmjopen.bmj.com/site/about/guidelines.xhtml

BMJ Open

Changes in medical student attendance and its impact on student educational outcomes – A systematic review protocol

Journal:	*BMJ Open*
Manuscript ID	bmjopen-2023-078252.R1
Article Type:	Protocol
Date Submitted by the Author:	09-Oct-2023
Complete List of Authors:	Nagappan, Palaniappan Ganesh; University of Cambridge, School of Clinical Medicine<br>Brown, Samuel; University of Cambridge<br>McManus, Alex; University of Cambridge<br>Sayers, Sarah; University of Cambridge,<br>Absar, Shazia; University of Cambridge<br>Tan, Sapphire Rou Xi; University of Cambridge<br>Kuhn, Isla; University of Cambridge<br>Tulinius, Charlotte; University of Cambridge<br>Lau, Edward; University of Cambridge
**Primary Subject Heading**:	Medical education and training
Secondary Subject Heading:	Communication, Ethics, Evidence based practice, Health policy
Keywords:	EDUCATION & TRAINING (see Medical Education & Training), Health Education, MEDICAL EDUCATION & TRAINING

SCHOLARONE™
Manuscripts

Changes in medical student attendance and its impact on student educational outcomes – A systematic review protocol

Contents

Administrative information 3
Title 3
Authors 3
Abstract 3
Introduction 3
Methods and analysis 4
Ethics and dissemination 4
Article Summary 4
Strengths and limitations of this study 4
Keywords 4
Introduction 4
Rationale 4
Research Question 5
Objectives 5
Methods 6
Eligibility criteria 6
Information sources 6
Search strategy 6
Study records 6
Data management 6
Selection process 6

For peer review only

Data collection process 7
Data items 7
Outcomes and prioritization 7
Risk of bias in individual studies 8
Data synthesis 8
Meta-bias(es) 8
Confidence in cumulative evidence 8
Ethics and dissemination 9
Patient and Public Involvement 9
Author Contributions 9
Funding 9
Competing interests' statement 9
References 10
Supplementary material 11
Search Strategies 11
Medline via Ovid 11
Embase via Ovid 11
Web of Science 11
Scopus 12
British Education Index via EBSCOhost 12
ERIC via EBSCOhost 13

For peer review only

For peer review only - http://bmjopen.bmj.com/site/about/guidelines.xhtml

Administrative information

Title

Changes in medical student attendance and its impact on student educational outcomes – A systematic review protocol

Authors

Dr Palaniappan Ganesh Nagappan. University of Cambridge: pgn25@cam.ac.uk

Mr Samuel Brown. University of Cambridge: sb2382@cam.ac.uk

Mr Alex McManus. University of Cambridge: am2776@cam.ac.uk

Miss Sarah Sayers. University of Cambridge: sbs51@cam.ac.uk

Miss Shazia Absar. University of Cambridge: sha36@cam.ac.uk

Dr Sapphire Tan Rou Xi, University of Cambridge: rxst2@cam.ac.uk

Miss Isla Kuhn. University of Cambridge: ilk21@cam.ac.uk

Dr Charlotte Tulinius. University of Cambridge: ct419@medschl.cam.ac.uk

Dr Edward Lau. University of Cambridge: ejsl2@cam.ac.uk

Corresponding author:

Dr Palaniappan Ganesh Nagappan
Emmanuel College
Cambridge, CB23AP, UK
+447472659583
Pgn25@cam.ac.uk

Word Count: 1921 words

Abstract

Introduction

The COVID-19 pandemic has had a significant impact on medical education, with many institutions shifting to online learning to ensure the safety of students and staff. However, there has been a decline in in-person attendance at medical schools across the UK and worldwide following the relaxation of social distancing rules and the reinstation of in-person teaching. Whilst reflected within the literature, there is currently no systematic review describing these changes. We aim to find out how medical students' attendance is changing as documented within the literature and its impact on their educational outcomes.

For peer review only

For peer review only - http://bmjopen.bmj.com/site/about/guidelines.xhtml

Methods and analysis

This systematic review will follow the guidelines of the Centre of Research and Dissemination, Moose, and PRISMA. We will search the major databases of Medline via Ovid, Embase via Ovid, Scopus, Web of Science, British Education Index via EBSCOhost and ERIC via EBSCOhost.

Two reviewers will independently screen each paper and extract data, with a third reviewer for dispute resolution. All studies reporting on medical students from various universities, both graduate and undergraduate, and describing changes in attendance and/or students' educational outcomes will be included. Risk of bias in individual studies will be assessed using the Newcastle-Ottawa Scale and confidence in cumulative evidence will be evaluated using the GRADE-CERQual approach. A narrative synthesis of the findings from all included studies will be reported.

Ethics and dissemination

Ethical approval is not required for this systematic review of existing publicly available literature.

Article Summary

Strengths and limitations of this study

- The study's adherence to comprehensive methodology, following established guidelines like the Centre of Research and Dissemination, Moose, and PRISMA, ensures a systematic and rigorous approach, enhancing credibility and reliability.
- The inclusion of diverse research types (qualitative, quantitative, mixed methods) provides a comprehensive understanding, allowing triangulation of evidence and strengthening the validity of conclusions.
- Independent data management and extraction by two pairs of reviewers reduce biases and enhance objectivity, leading to more robust and credible research outcomes.
- Being a retrospective, observational research design, systematic reviews are subject to systematic and random error. We hope to minimise this by strict adherence to the guidelines mentioned above.
- Potential language bias could lead to oversight of relevant studies, and addressing these limitations is essential for a more comprehensive analysis.

Keywords

- Medical Students (MeSH Term: Students, Medical)
- Attendance (MeSH Term: Absenteeism)
- Performance (MeSH Term: Academic Performance)

For peer review only - http://bmjopen.bmj.com/site/about/guidelines.xhtml

Introduction

Rationale

Post-pandemic, following a shift back to in-person teaching, the level of medical student attendance at many higher education institutions, including medical schools across the UK seems to have fallen dramatically. Teachers and leads across all subjects report a very low attendance rate at small group and larger group teaching and whole cohort lectures and a similar phenomenon has been reported across other medical schools in the UK. Although the literature is still limited on this development, this appears to be a worldwide development (1).

Before the pandemic, most students would have expected to be on clinical placements all day, but the pandemic introduced the need for replacement of in-person education with creative experiments involving learning online and from home, and the application of telemedicine (2). This need acted as a catalyst and accelerated the development of online learning. With the shift back to in-person teaching, some students are now actively opting out of in-person teaching (3). This could be explained by students realising that attending core curriculum teaching in person is no longer an absolute necessity for successful completion of the curriculum (1), though there may be other reasons aside from just the availability of online learning leading to the observed drop in attendance at teaching sessions. A greater understanding of what these changes in attendance are, along with why the changes in attendance are happening and its impact on student educational outcomes would be valuable in determining whether further action is required to maintain high standards of medical education.

Identifying areas where institutions can help support this evolving learning process is essential to continue to provide a holistic education. Without this, students' learning may be at risk of becoming overtly focused in some areas and less in others. Higher education online learning was available before the COVID-19 pandemic but this was often variable and delivered asynchronously (4). Comparing previously adopted teaching strategies and their impact on student educational outcomes can help to guide the development of innovative models of teaching to ensure continuous high-quality education.

To date, there are no systematic reviews published that explore the changes in attendance documented within the literature, and its subsequent influence on student education outcomes. This systematic review aims to describe and explain the changes in medical student attendance and explore the potential impacts this may have on student educational outcomes.

Research Question

Research question: in what ways is student attendance to in-person teaching sessions changing and what are the impacts of this on student educational outcomes?

Objectives

Primary aim

- To describe the changes in medical student attendance at in-person teaching sessions

Secondary aims

For peer review only - http://bmjopen.bmj.com/site/about/guidelines.xhtml

- To explain the changes to medical student attendance at in-person teaching sessions.
- To describe the impact of the changes in attendance on medical student educational outcomes.
- To describe strategies adopted by educators in light of these changes to medical student attendance at in-person teaching sessions.
- To describe the impact of the COVID-19 pandemic on medical student attendance for in-person teaching sessions.

Methods

The design and methods used for this systematic review comply with Centre of Research and Dissemination Guidelines, Meta-analyses of Observational Studies in Epidemiology (Moose) and are reported in line with Preferred Reporting Items for Systematic Reviews and Meta-Analyses (PRISMA). Eligibility criteria were informed using the SPIDER and MOOSE guidelines. This protocol adheres to the PRISMA-P guidelines ("Supplementary Material 1.pdf").

Eligibility criteria

(S) Sample: Medical students in both graduate and undergraduate medical curriculums across all types of universities (private and public)

(PI) The phenomenon of Interest: Changes in attendance, educational outcomes, or both.

(E) Evaluation: 1. Recorded or anecdotal evidence of changes in attendance and 2. Comparison between 2 or more sets of examination scores or any other performance-based measures (including interest, satisfaction and confidence rates).

(D) Design: Primary studies excluding grey literature.

(R) Research type: Primary studies of qualitative, quantitative and mixed-methods research could be searched for, not including systematic reviews, literature reviews or metanalysis.

Information sources

The search will employ topic-based strategies designed for each database from inception to 20th September 2023. There will be no language or geographical restrictions.

Databases:

EMBASE via OVID, MEDLINE via OVID, Scopus, Web of Science Core Collection, British Education Index via EBSCOhost, ERIC via EBSCOhost

Search strategy

The detailed search strategies for the respective databases can be found under Supplementary Materials ("Supplementary Material 2.pdf").

For peer review only - http://bmjopen.bmj.com/site/about/guidelines.xhtml

For peer review only

Study records

Data management

Records will be managed through Endnote and NVIVO.

Selection process

Inclusion criteria:

- Medical students studying in medical schools accredited by their country's governing body.
- Language of Publication: all languages
- Settings: Hospitals, medical schools
- At least one of the following outcomes: 1. attendance rates, 2. performance measures, and 3. solutions.

Exclusion criteria:

- Grey literature
- Secondary research

The screening will be performed independently by two pairs of reviewers (SS/SA; SB/AM, with each pair covering 50% of the papers, with a third individual to resolve any disagreements.

Data collection process

Using a standardised form, two pairs of reviewers (SS/SA; SB/AM) will extract the data independently, with each pair covering 50% of the papers. A third reviewer (PN) will independently check the data for consistency and clarity.

Data items

The data extracted will include the following summary data:

- Sample characteristics
- Sample size
- Study date
- Medium of lecture / programme / teaching
- Stage of medical education training
- Academic outcome / performance scores
- Academic outcome / performance measure used
- Confidence, interest and satisfaction rates
- Attendance rates (stratified by in-person and otherwise)
- Access rates to online materials
- Solutions / interventions used
- Association between attendance rates and academic outcomes / performance

For peer review only - http://bmjopen.bmj.com/site/about/guidelines.xhtml

- Students' perception
- Other outcomes

This will be in addition to the critical arguments raised during the process. Both qualitative and quantitative data will be collected for narrative synthesis.

Outcomes and prioritization

1. Recorded or anecdotal evidence of changes in attendance at live learning opportunities both in person and otherwise. This would include formative and summative assessments as well if it is used as a "technique" for learning rather than only as a measurement of performance. An example of an in-person teaching assessment is an OSCE setup.

2. Comparison of examination scores, performance-based measures, or educational outcomes with the implementation of a learning technique or intervention.

Risk of bias in individual studies

The risk of bias for each included trial will be independently assessed by the same initial reviewers. The fifth reviewer will mediate in situations of disagreement. Cohen's κ will be used to assess agreement between reviewers. All tools and processes will be piloted before use. The risk of bias will be assessed using the Newcastle-Ottawa Scale (5,6).

The following assessments will then be made, depending on the study type:

Qualitative studies: The reviewers will assess the appropriateness of data sources and analytical processes, the study's transparency, consideration of context/setting, and the study's consideration of researchers' reflexivity and positioning.

Descriptive cross-sectional quantitative studies: The reviewers will assess the appropriateness of the study's sampling strategy and the representativeness of the sample, the use of appropriate measurement instruments, and the acceptability of the response rate.

Nonrandomised quantitative studies: The reviewers will assess the study's minimisation of selection bias, use of appropriate measurement instruments, use of comparable groups across study conditions, and the completeness of outcome data.

Data synthesis

Qualitative Data: Qualitative data will be imported into VIVO software. Malterud's systematic text condensation as thematic analysis will be conducted by two reviewers independently with a discussion following this. (7–10)

Quantitative Data: The data collected will undergo narrative synthesis. A meta-analysis will be considered should the quantitative data be sufficiently homogenous.

For peer review only - http://bmjopen.bmj.com/site/about/guidelines.xhtml

Meta-bias(es)

N/A

Confidence in cumulative evidence

Confidence in discrete review findings will be assessed using the recently developed Grading of Recommendations, Assessment, Development and Evaluation - Confidence in the Evidence from Reviews of Qualitative Research (GRADE-CERQual) (11). Assessment of confidence in a given review finding involves evaluating how likely it is that the finding represents a real phenomenon, i.e. factors leading to changes in attendance, performance and their relationship with each other. This assessment will be based upon an evaluation of the following: (i) methodological limitations of the primary studies contributing to the finding, (ii) the relevance of the primary contributing studies regarding the objectives of the systematic review, (iii) the coherence of the finding, and (iv) the adequacy of data supporting the finding.

A summary table will list each review finding—primary contributing studies, evaluations of the above four domains, an overall confidence rating (high, moderate, low, or very low), and an explanation of the rating judgement.

Ethics and dissemination

Ethical approval is not required for this systematic review of existing publicly available literature.

Patient and Public Involvement

None.

Author Contributions

PGN: Drafted the proposal and conceptualisation of the topic.

SB: Data collection and refining of the topic.

AM: Data collection and refining of the topic.

SS: Data collection and refining of the topic.

SA: Data collection and refining of the topic.

ST: Conceptualisation of the topic.

IK: Reviewing and critiquing to improve research methodology with the drafting of the search strategy.

CT: Conceptualisation of topic and supervision.

For peer review only - http://bmjopen.bmj.com/site/about/guidelines.xhtml

EL: Conceptualisation of topic and supervision.

Funding

This research received no specific grant from any funding agency in the public, commercial or not-for-profit sectors. However, the necessary NVivo licenses will be available from the University of Cambridge.

Competing interests' statement

There are no known competing interests.

For peer review only

References

1. Gardner G, Feldman M, Santen SA, Mui P, Biskobing D. Determinants and Outcomes of In-person Lecture Attendance in Medical School. Med Sci Educ. 2022 Aug;32(4):883–90.

2. Park H, Shim S, Lee YM. A scoping review on adaptations of clinical education for medical students during COVID-19. Prim Care Diabetes. 2021 Dec;15(6):958–76.

3. Jhajj S, Kaur P, Jhajj P, Ramadan A, Jain P, Upadhyay S, et al. Impact of Covid-19 on Medical Students around the Globe. J Community Hosp Intern Med Perspect. 2022;12(4):1–6.

4. Tallent-Runnels MK, Thomas JA, Lan WY, Cooper S, Ahern TC, Shaw SM, et al. Teaching Courses Online: A Review of the Research. Review of Educational Research. 2006 Mar 1;76(1):93–135.

5. Deeks JJ, Dinnes J, D'Amico R, Sowden AJ, Sakarovitch C, Song F, et al. Evaluating non-randomised intervention studies. Health technology assessment (Winchester, England). 2003;7(27):iii–173.

6. Downs SH, Black N. The feasibility of creating a checklist for the assessment of the methodological quality both of randomised and non-randomised studies of health care interventions. J Epidemiol Community Health. 1998 Jun;52(6):377–84.

7. Malterud K. Systematic text condensation: a strategy for qualitative analysis. Scand J Public Health. 2012 Dec;40(8):795–805.

8. Dixon-Woods M, Cavers D, Agarwal S, Annandale E, Arthur A, Harvey J, et al. Conducting a critical interpretive synthesis of the literature on access to healthcare by vulnerable groups. BMC Medical Research Methodology. 2006 Jul 26;6(1):35.

9. Entwistle V, Firnigl D, Ryan M, Francis J, Kinghorn P. Which experiences of health care delivery matter to service users and why? A critical interpretive synthesis and conceptual map. J Health Serv Res Policy. 2012 Apr;17(2):70–8.

10. Schick-Makaroff K, MacDonald M, Plummer M, Burgess J, Neander W. What Synthesis Methodology Should I Use? A Review and Analysis of Approaches to Research Synthesis. AIMS Public Health. 2016;3(1):172–215.

For peer review only - http://bmjopen.bmj.com/site/about/guidelines.xhtml

11. Lewin S, Booth A, Glenton C, Munthe-Kaas H, Rashidian A, Wainwright M, et al. Applying GRADE-CERQual to qualitative evidence synthesis findings: introduction to the series. Implementation Science. 2018 Jan 25;13(1):2.

For peer review only

For peer review only - http://bmjopen.bmj.com/site/about/guidelines.xhtml

Supplementary Material 1

PRISMA-P Checklist

PRISMA-P (Preferred Reporting Items for Systematic review and Meta-Analysis Protocols) 2015 checklist

Section and topic	Item No	Checklist item	Line numbers
**ADMINISTRATIVE INFORMATION**			
Title:			
Identification	1a	Identify the report as a protocol of a systematic review	1-3
Update	1b	If the protocol is for an update of a previous systematic review, identify as such	n/a
Registration	2	If registered, provide the name of the registry (such as PROSPERO) and registration number	n/a
Authors:			
Contact	3a	Provide name, institutional affiliation, e-mail address of all protocol authors; provide physical mailing address of corresponding author	53-69
Contributions	3b	Describe contributions of protocol authors and identify the guarantor of the review	278-287
Amendments	4	If the protocol represents an amendment of a previously completed or published protocol, identify as such and list changes	n/a
Support:			
Sources	5a	Indicate sources of financial or other support for the review	289-291
Sponsor	5b	Provide name for the review funder and/or sponsor	289-291
Role of sponsor or funder	5c	Describe roles of funder(s), sponsor(s), and/or institution(s), if any, in developing the protocol	289-291
**INTRODUCTION**			
Rationale	6	Describe the rationale for the review in the context of what is already known	116-143
Objectives	7	Provide an explicit statement of the question(s) the review will address with reference to participants, interventions, comparators, and outcomes (PICO)	144-146
**METHODS**			
Eligibility criteria	8	Specify the study characteristics (such as PICO, study design, setting, time frame) and report characteristics (such as years considered, language, publication status) to be used as criteria for eligibility for the review	164-173

For peer review only

Information sources	9	Describe all intended information sources (such as electronic databases, contact with study authors, trial registers or other grey literature sources) with planned dates of coverage	175-180
Search strategy	10	Present draft of search strategy to be used for at least one electronic database, including planned limits, such that it could be repeated	Supplementary material 1.pdf
Study records:			
Data management	11a	Describe the mechanism(s) that will be used to manage records and data throughout the review	186-188
Selection process	11b	State the process that will be used for selecting studies (such as two independent reviewers) through each phase of the review (that is, screening, eligibility and inclusion in meta-analysis)	197-198
Data collection process	11c	Describe planned method of extracting data from reports (such as piloting forms, done independently, in duplicate), any processes for obtaining and confirming data from investigators	201-205
Data items	12	List and define all variables for which data will be sought (such as PICO items, funding sources), any pre-planned data assumptions and simplifications	207-222
Outcomes and prioritization	13	List and define all outcomes for which data will be sought, including prioritization of main and additional outcomes, with rationale	226-232
Risk of bias in individual studies	14	Describe anticipated methods for assessing risk of bias of individual studies, including whether this will be done at the outcome or study level, or both; state how this information will be used in data synthesis	234-248
Data synthesis	15a	Describe criteria under which study data will be quantitatively synthesised	250-255
	15b	If data are appropriate for quantitative synthesis, describe planned summary measures, methods of handling data and methods of combining data from studies, including any planned exploration of consistency (such as I^2, Kendall's τ)	254-255
	15c	Describe any proposed additional analyses (such as sensitivity or subgroup analyses, meta-regression)	252-253
	15d	If quantitative synthesis is not appropriate, describe the type of summary planned	251-253
Meta-bias(es)	16	Specify any planned assessment of meta-bias(es) (such as publication bias across studies, selective reporting within studies)	257-258
Confidence in cumulative evidence	17	Describe how the strength of the body of evidence will be assessed (such as GRADE)	260-272

For peer review only

It is strongly recommended that this checklist be read in conjunction with the PRISMA-P Explanation and Elaboration for important clarification on the items. Amendments to a review protocol should be tracked and dated. The copyright for PRISMA-P (including checklist) is held by the PRISMA-P Group and is distributed under a Creative Commons Attribution Licence 4.0.

From: Shamseer L, Moher D, Clarke M, Ghersi D, Liberati A, Petticrew M, Shekelle P, Stewart L, PRISMA-P Group. Preferred reporting items for systematic review and meta-analysis protocols (PRISMA-P) 2015: elaboration and explanation. BMJ. 2015 Jan 2;349(jan02 1):g7647.

For peer review only - http://bmjopen.bmj.com/site/about/guidelines.xhtml

Supplementary Material 2

Search Strategies

Medline via Ovid

Ovid MEDLINE(R) and Epub Ahead of Print, In-Process, In-Data-Review & Other Non-Indexed Citations, Daily and Versions <1946 to September 20, 2023>		
1	((clinical or medical or doctor) adj2 (student* or trainee* or school*)).ti,ab.	90738
2	exp Schools, Medical/ or exp Students, Medical/ or exp education, medical, undergraduate/	80136
3	1 or 2	126117
4	(Absen* or non-attendance or nonattendance or attend*).ti,ab.	1079900
5	(Placemen* or Lectur* or Semina* or online or virtual or teaching or "in person" or inperson or "face to face" or traditional or class*).ti,ab.	2761182
6	exp Teaching/	93309
7	5 or 6	2811415
8	(Exam* or Perform* or Attainment* or Result*).ti,ab.	15523220
9	exp academic performance/ or exp educational status/ or exp academic success/	65300
10	8 or 9	15540736
11	3 and 4 and 7 and 10	3160

Embase via Ovid

Embase <1974 to 2023 September 20>		
1	((clinical or medical or doctor) adj2 (student* or trainee* or school*)).ti,ab. or exp *medical school/ or exp *medical student/	131819
2	(Absen* or non-attendance or nonattendance or attend*).ti,ab. or exp *school attendance/	1459034
3	(Placemen* or Lectur* or Semina* or online or virtual or teaching or "in person" or inperson or "face to face" or traditional or class*).ti,ab. or exp *Teaching/	3657834
4	exp academic achievement/ or exp *educational status/ or (Exam* or Perform* or Attainment* or Result*).ti,ab.	20509010
5	1 and 2 and 3 and 4	5027
6	limit 5 to embase	1697

Web of Science

Web of Science Search Strategy (v0.1)
Database: Web of Science Core Collection
Entitlements:
- WOS.IC: 1993 to 2023
- WOS.CCR: 1985 to 2023
- WOS.SCI: 1900 to 2023
- WOS.AHCI: 1975 to 2023
- WOS.BHCI: 2008 to 2023
- WOS.BSCI: 2008 to 2023

For peer review only

- WOS.ESCI: 2018 to 2023<br>- WOS.ISTP: 1990 to 2023<br>- WOS.SSCI: 1956 to 2023<br>- WOS.ISSHP: 1990 to 2023<br>Date Run: Thu Sep 20, 2023		
1	TS=(Absen* OR "non-attendance" OR attendance)	1135903
2	TS= ((clinical OR medical OR doctor) near/2 (student* OR trainee* OR school*))	97987
3	TS=(Placemen* OR Lectur* OR Semina* OR online OR virtual OR teaching or "in person" or inperson or "face to face" or traditional or class*)	6854033
4	TS=(Exam* OR Perform* OR Attainment* OR Result* OR "academic performance" OR "educational status")	6854033
5	TS=(Exam* OR Perform* OR Attainment* OR Result* OR "academic performance" OR "educational status")	29046391
6	#4 AND #3 AND #2 AND #1	811

Scopus

Scopus<br>Date Run: Thu Sep 20, 2023	
1	TITLE-ABS-KEY (((absen* OR "non-attend*" OR attend* OR nonattend*) AND ((clinical OR medical OR doctor) W/2 (student* OR trainee* OR school*)) AND (placemen* OR lectur* OR semina* OR online OR virtual OR teaching OR "in person" OR inperson OR "face to face" OR traditional OR class*) AND (exam* OR perform* OR attainment* OR result* OR "academic performance" OR "educational status")))

British Education Index via EBSCOhost

Interface - EBSCOhost Research Databases<br>Search Screen - Advanced<br>Search Database - British Education Index<br><br>Expanders - Apply equivalent subjects<br>Search modes - Boolean/Phrase<br>Date Run: Thu Sep 20, 2023		
S1	TI ((clinical or medical or doctor) w2 (student* or trainee* or school*)) OR AB ((clinical or medical or doctor) w2 (student* or trainee* or school*))	4,076
S2	((DE "MEDICAL schools") OR (DE "MEDICAL students") OR (DE "MEDICAL education"))	9,405
S3	S1 OR S2	10,242
S4	TI ((Absen* or non-attendance or nonattendance or attend*)) OR ((Absen* or non-attendance or nonattendance or attend*))	7,649

For peer review only - http://bmjopen.bmj.com/site/about/guidelines.xhtml

S5	DE "SCHOOL attendance"	610
S6	S4 OR S5	7,649
S7	TI ((Placemen* or Lectur* or Semina* or online or virtual or teaching or "in person" or inperson or "face to face" or traditional or class*)) OR AB ((Placemen* or Lectur* or Semina* or online or virtual or teaching or "in person" or inperson or "face to face" or traditional or class*))	76,866
S8	DE "TEACHING"	6,709
S9	S7 OR S8	79,470
S10	TI ((Exam* or Perform* or Attainment* or Result*)) OR AB ((Exam* or Perform* or Attainment* or Result*))	80,327
S11	(DE "ACADEMIC achievement") OR (DE "EDUCATIONAL attainment")	10,645
S12	S10 OR S11	85,240
S13	S3 AND S6 AND S9 AND S12	101

For peer review only

ERIC via EBSCOhost

Interface - EBSCOhost Research Databases
Search Screen - Advanced
Search Database - ERIC

Expanders - Apply equivalent subjects
Search modes - Boolean/Phrase
Date Run: Thu Sep 20, 2023

S1	(AB (clinical or medical or doctor) w2 (student* or trainee* or school*)) OR (TI (clinical or medical or doctor) w2 (student* or trainee* or school*))	7,784
S2	TI (DE "Medical Schools") OR (DE "Medical Students") OR (DE "Medical Education")	397
S3	S1 OR S2	7,842
S4	(AB (Absen* or non-attendance or nonattendance or attend*)) OR (TI (Absen* or non-attendance or nonattendance or attend*))	73,324
S5	(AB (Placemen* or Lectur* or Semina* or online or virtual or teaching or "in person" or inperson or "face to face" or traditional or class*)) OR (TI (Placemen* or Lectur*	672,070

For peer review only - http://bmjopen.bmj.com/site/about/guidelines.xhtml

	or Semina* or online or virtual or teaching or "in person" or inperson or "face to face" or traditional or class*))	
S6	DE "Teaching Methods"	211,764
S7	S5 OR S6	737,112
S8	(AB (Exam* or Perform* or Attainment* or Result*)) OR (TI (Exam* or Perform* or Attainment* or Result*))	845,630
S9	DE "Academic Achievement"	94,882
S10	S8 OR S9	876,868
S11	S3 AND S4 AND S7 AND S10	138

13

For peer review only

For peer review only - http://bmjopen.bmj.com/site/about/guidelines.xhtml

BMJ Open

Changes in medical student attendance and its impact on student educational outcomes – A systematic review protocol

Journal:	*BMJ Open*
Manuscript ID	bmjopen-2023-078252.R2
Article Type:	Protocol
Date Submitted by the Author:	28-Nov-2023
Complete List of Authors:	Nagappan, Palaniappan Ganesh; University of Cambridge, School of Clinical Medicine<br>Brown, Samuel; University of Cambridge<br>McManus, Alex; University of Cambridge<br>Sayers, Sarah; University of Cambridge,<br>Absar, Shazia; University of Cambridge<br>Tan, Sapphire Rou Xi; University of Cambridge<br>Kuhn, Isla; University of Cambridge<br>Tulinius, Charlotte; University of Cambridge<br>Lau, Edward; University of Cambridge
**Primary Subject Heading**:	Medical education and training
Secondary Subject Heading:	Communication, Ethics, Evidence based practice, Health policy
Keywords:	EDUCATION & TRAINING (see Medical Education & Training), Health Education, MEDICAL EDUCATION & TRAINING

SCHOLARONE™
Manuscripts

Changes in medical student attendance and its impact on student educational outcomes – A systematic review protocol

For peer review only

Contents

Administrative information 3
- Title 3
- Authors 3

Abstract 3
- Introduction 3
- Methods and analysis 4
- Ethics and dissemination 4

Article Summary 4
- Strengths and limitations of this study 4
- Keywords 4

Introduction 4
- Rationale 4

Research Question 5
- Objectives 5

Methods 6
- Eligibility criteria 6
- Information sources 6
- Search strategy 6
- Study records 6
  - Data management 6
  - Selection process 6

For peer review only - http://bmjopen.bmj.com/site/about/guidelines.xhtml

Data collection process 7
Data items 7
Outcomes and prioritization 7
Risk of bias in individual studies 8
Data synthesis 8
Meta-bias(es) 8
Confidence in cumulative evidence 8
Ethics and dissemination 9
Patient and Public Involvement 9
Author Contributions 9
Funding 9
Competing interests' statement 9
References 10
Supplementary material 11
Search Strategies 11
Medline via Ovid 11
Embase via Ovid 11
Web of Science 11
Scopus 12
British Education Index via EBSCOhost 12
ERIC via EBSCOhost 13

For peer review only

For peer review only - http://bmjopen.bmj.com/site/about/guidelines.xhtml

Administrative information

Title

Changes in medical student attendance and its impact on student educational outcomes – A systematic review protocol

Authors

Dr Palaniappan Ganesh Nagappan. University of Cambridge: pgn25@cam.ac.uk

Mr Samuel Brown. University of Cambridge: sb2382@cam.ac.uk

Mr Alex McManus. University of Cambridge: am2776@cam.ac.uk

Miss Sarah Sayers. University of Cambridge: sbs51@cam.ac.uk

Miss Shazia Absar. University of Cambridge: sha36@cam.ac.uk

Dr Sapphire Tan Rou Xi, University of Cambridge: rxst2@cam.ac.uk

Miss Isla Kuhn. University of Cambridge: ilk21@cam.ac.uk

Dr Charlotte Tulinius. University of Cambridge: ct419@medschl.cam.ac.uk

Dr Edward Lau. University of Cambridge: ejsl2@cam.ac.uk

Corresponding author:

Dr Palaniappan Ganesh Nagappan
Emmanuel College
Cambridge, CB23AP, UK
+447472659583
Pgn25@cam.ac.uk

Word Count: 1921 words

Abstract

Introduction

The COVID-19 pandemic has had a significant impact on medical education, with many institutions shifting to online learning to ensure the safety of students and staff. However, there has been a decline in in-person attendance at medical schools across the UK and worldwide following the relaxation of social distancing rules and the reinstation of in-person teaching. Whilst reflected within the literature, there is currently no systematic review describing these changes. We aim to find out how medical students' attendance is changing as documented within the literature and its impact on their educational outcomes.

For peer review only

Methods and analysis

This systematic review will follow the guidelines of the Centre of Research and Dissemination, Moose, and PRISMA. We will search the major databases of Medline via Ovid, Embase via Ovid, Scopus, Web of Science, British Education Index via EBSCOhost and ERIC via EBSCOhost.

Two reviewers will independently screen each paper and extract data, with a third reviewer for dispute resolution. All studies reporting on medical students from various universities, both graduate and undergraduate and describing changes in attendance and/or students' educational outcomes will be included. Risk of bias in individual studies will be assessed using the Newcastle-Ottawa Scale and confidence in cumulative evidence will be evaluated using the GRADE-CERQual approach. A narrative synthesis of the findings from all included studies will be reported.

Ethics and dissemination

Ethical approval is not required for this systematic review of existing publicly available literature. We will subsequently aim to publish the results of this systematic review in a peer-reviewed journal.

Article Summary

Strengths and limitations of this study

- The study's adherence to comprehensive methodology, following established guidelines like the Centre of Research and Dissemination, Moose, and PRISMA, ensures a systematic and rigorous approach, enhancing credibility and reliability.
- The inclusion of diverse research types (qualitative, quantitative, mixed methods) provides a comprehensive understanding, allowing triangulation of evidence and strengthening the validity of conclusions.
- Independent data management and extraction by two pairs of reviewers reduce biases and enhance objectivity, leading to more robust and credible research outcomes.
- Being a retrospective, observational research design, systematic reviews are subject to systematic and random error. We hope to minimise this by strict adherence to the guidelines mentioned above.
- Potential language bias could lead to oversight of relevant studies, and addressing these limitations is essential for a more comprehensive analysis.

Keywords

- Medical Students (MeSH Term: Students, Medical)
- Attendance (MeSH Term: Absenteeism)
- Performance (MeSH Term: Academic Performance)

For peer review only - http://bmjopen.bmj.com/site/about/guidelines.xhtml

Introduction

Rationale

Post-pandemic, following a shift back to in-person teaching, the level of medical student attendance at many higher education institutions, including medical schools across the UK seems to have fallen dramatically. Teachers and leads across all subjects report a very low attendance rate at small group and larger group teaching and whole cohort lectures and a similar phenomenon has been reported across other medical schools in the UK. Although the literature is still limited on this development, this appears to be a worldwide observation (1). At our institution and within the literature, this change in the attendance was noted to occur prior to the pandemic (2), but became significantly more noticeable following the return to in-person teaching post-pandemic.

Before the COVID-19 pandemic, most students would have expected to be on clinical placements all day, but the pandemic introduced the need for replacement of in-person education with creative experiments involving learning online and from home, and the application of telemedicine (3). This need acted as a catalyst and accelerated the development of online learning. With the shift back to in-person teaching, some students are now actively opting out of in-person teaching (4). Whilst we acknowledge that the COVID-19 pandemic may have been a factor contributing to this observation, as this was noted prior to the return to in-person teaching, there may be other explanations and the changes in attendance may reflect a shift in the direction of medical education, raising the question of whether attendance is a good measure of high quality medical education (5). For example, this could be explained by students increasingly realising that attending core curriculum teaching in person is no longer an absolute necessity for successful completion of the curriculum (1) or an increase in the utility and availability of online learning materials. A greater understanding of what these changes in attendance are, along with why the changes in attendance are happening and its impact on student educational outcomes would be valuable in determining whether further action is required to maintain high standards of medical education.

Identifying areas where institutions can help support this evolving learning process is essential to continue to provide a holistic education. Without this, students' learning may be at risk of becoming overtly focused in some areas and less in others. Higher education online learning was available before the COVID-19 pandemic but this was often variable and delivered asynchronously (6). Exploring previously adopted teaching strategies and their impact on student educational outcomes can help to guide the development of innovative models of teaching to ensure continuous high-quality education.

To date, there are no systematic reviews published that describe the changes in attendance documented within the literature, and its subsequent influence on student education outcomes. This systematic review aims to explore and explain the ongoing changes in medical student attendance that have been noted to start prior to but exacerbated by the pandemic and describe the potential impacts this may have on student educational outcomes.

Research Question

Research question: in what ways is student attendance to in-person teaching sessions changing and what are the impacts of this on student educational outcomes?

For peer review only - http://bmjopen.bmj.com/site/about/guidelines.xhtml

Objectives

Primary aim

- To describe the changes in medical student attendance at in-person teaching sessions

Secondary aims

- To explain the changes to medical student attendance at in-person teaching sessions.
- To describe the impact of the changes in attendance on medical student educational outcomes.
- To describe strategies adopted by educators in light of these changes to medical student attendance at in-person teaching sessions.

Methods

The design and methods used for this systematic review comply with Centre of Research and Dissemination Guidelines, Meta-analyses of Observational Studies in Epidemiology (Moose) and are reported in line with Preferred Reporting Items for Systematic Reviews and Meta-Analyses (PRISMA). Eligibility criteria were informed using the SPIDER and MOOSE guidelines. This protocol adheres to the PRISMA-P guidelines ("Supplementary Material 1.pdf").

Eligibility criteria

(S) Sample: Medical students in both graduate and undergraduate medical curriculums across all types of universities (private and public)

(PI) The phenomenon of Interest: Changes in attendance, educational outcomes, or both.

(E) Evaluation: 1. Recorded or anecdotal evidence of changes in attendance and 2. Comparison between 2 or more sets of examination scores or any other performance-based measures (including interest, satisfaction and confidence rates).

(D) Design: Primary studies excluding grey literature.

(R) Research type: Primary studies of qualitative, quantitative and mixed-methods research could be searched for, not including systematic reviews, literature reviews or metanalysis.

Information sources

The search will employ topic-based strategies designed for each database from inception to 20th September 2023. There will be no language or geographical restrictions.

Databases:

EMBASE via OVID, MEDLINE via OVID, Scopus, Web of Science Core Collection, British Education Index via EBSCOhost, ERIC via EBSCOhost

For peer review only - http://bmjopen.bmj.com/site/about/guidelines.xhtml

Search strategy

The detailed search strategies for the respective databases can be found under Supplementary Materials ("Supplementary Material 2.pdf").

Study records

Data management

Records will be managed through Endnote and NVIVO.

Selection process

Inclusion criteria:

- Medical students studying in medical schools accredited by their country's governing body.
- Language of Publication: all languages
- Settings: Hospitals, medical schools
- At least one of the following outcomes: 1. attendance rates, 2. performance measures, and 3. solutions.

Exclusion criteria:

- Grey literature
- Secondary research

The screening will be performed independently by two pairs of reviewers (SS/SA; SB/AM, with each pair covering 50% of the papers, with a third individual to resolve any disagreements.

Data collection process

Using a standardised form, two pairs of reviewers (SS/SA; SB/AM) will extract the data independently, with each pair covering 50% of the papers. A third reviewer (PN) will independently check the data for consistency and clarity.

Data items

The data extracted will include the following summary data:

- Sample characteristics
- Sample size
- Study date
- Medium of lecture / programme / teaching
- Stage of medical education training
- Academic outcome / performance scores

For peer review only - http://bmjopen.bmj.com/site/about/guidelines.xhtml

- Academic outcome / performance measure used
- Confidence, interest and satisfaction rates
- Attendance rates (stratified by in-person and otherwise)
- Access rates to online materials
- Solutions / interventions used
- Association between attendance rates and academic outcomes / performance
- Students' perception
- Other outcomes

This will be in addition to the critical arguments raised during the process. Both qualitative and quantitative data will be collected for narrative synthesis.

Outcomes and prioritization

1. Recorded or anecdotal evidence of changes in attendance at live learning opportunities both in person and otherwise. This would include formative and summative assessments as well if it is used as a "technique" for learning rather than only as a measurement of performance. An example of an in-person teaching assessment is an OSCE setup.

2. Comparison of examination scores, performance-based measures, or educational outcomes with the implementation of a learning technique or intervention.

Risk of bias in individual studies

The risk of bias for each included trial will be independently assessed by the same initial reviewers. The fifth reviewer will mediate in situations of disagreement. Cohen's κ will be used to assess agreement between reviewers. All tools and processes will be piloted before use. The risk of bias will be assessed using the Newcastle-Ottawa Scale (7,8).

The following assessments will then be made, depending on the study type:

Qualitative studies: The reviewers will assess the appropriateness of data sources and analytical processes, the study's transparency, consideration of context/setting, and the study's consideration of researchers' reflexivity and positioning.

Descriptive cross-sectional quantitative studies: The reviewers will assess the appropriateness of the study's sampling strategy and the representativeness of the sample, the use of appropriate measurement instruments, and the acceptability of the response rate.

Nonrandomised quantitative studies: The reviewers will assess the study's minimisation of selection bias, use of appropriate measurement instruments, use of comparable groups across study conditions, and the completeness of outcome data.

For peer review only - http://bmjopen.bmj.com/site/about/guidelines.xhtml

Data synthesis

Qualitative Data: Qualitative data will be imported into VIVO software. Malterud's systematic text condensation as thematic analysis will be conducted by two reviewers independently with a discussion following this. (9–12)

Quantitative Data: The data collected will undergo narrative synthesis. A meta-analysis will be considered should the quantitative data be sufficiently homogenous.

Meta-bias(es)

N/A

Confidence in cumulative evidence

Confidence in discrete review findings will be assessed using the recently developed Grading of Recommendations, Assessment, Development and Evaluation - Confidence in the Evidence from Reviews of Qualitative Research (GRADE-CERQual) (13). Assessment of confidence in a given review finding involves evaluating how likely it is that the finding represents a real phenomenon, i.e. factors leading to changes in attendance, performance and their relationship with each other. This assessment will be based upon an evaluation of the following: (i) methodological limitations of the primary studies contributing to the finding, (ii) the relevance of the primary contributing studies regarding the objectives of the systematic review, (iii) the coherence of the finding, and (iv) the adequacy of data supporting the finding.

A summary table will list each review finding—primary contributing studies, evaluations of the above four domains, an overall confidence rating (high, moderate, low, or very low), and an explanation of the rating judgement.

Ethics and dissemination

Ethical approval is not required for this systematic review of existing publicly available literature. We will subsequently aim to publish the results of this systematic review in a peer-reviewed journal.

Patient and Public Involvement

None.

Author Contributions

PGN: Drafted the proposal and conceptualisation of the topic.

SB: Data collection and refining of the topic.

AM: Data collection and refining of the topic.

For peer review only - http://bmjopen.bmj.com/site/about/guidelines.xhtml

SS: Data collection and refining of the topic.

SA: Data collection and refining of the topic.

ST: Conceptualisation of the topic.

IK: Reviewing and critiquing to improve research methodology with the drafting of the search strategy.

CT: Conceptualisation of topic and supervision.

EL: Conceptualisation of topic and supervision.

Funding

This research received no specific grant from any funding agency in the public, commercial or not-for-profit sectors. However, the necessary NVivo licenses will be available from the University of Cambridge.

Competing interests' statement

There are no known competing interests.

References

1. Gardner G, Feldman M, Santen SA, Mui P, Biskobing D. Determinants and Outcomes of In-person Lecture Attendance in Medical School. Med Sci Educ. 2022 Aug;32(4):883–90.

2. Emahiser J, Nguyen J, Vanier C, Sadik A. Study of Live Lecture Attendance, Student Perceptions and Expectations. Med Sci Educ. 2021 Apr;31(2):697–707.

3. Park H, Shim S, Lee YM. A scoping review on adaptations of clinical education for medical students during COVID-19. Prim Care Diabetes. 2021 Dec;15(6):958–76.

4. Jhajj S, Kaur P, Jhajj P, Ramadan A, Jain P, Upadhyay S, et al. Impact of Covid-19 on Medical Students around the Globe. J Community Hosp Intern Med Perspect. 2022;12(4):1–6.

5. Kanter SL. To Be There or Not To Be There: Is Attendance Really the Question? Academic Medicine. 2012 Jun;87(6):679.

6. Tallent-Runnels MK, Thomas JA, Lan WY, Cooper S, Ahern TC, Shaw SM, et al. Teaching Courses Online: A Review of the Research. Review of Educational Research. 2006 Mar 1;76(1):93–135.

7. Deeks JJ, Dinnes J, D'Amico R, Sowden AJ, Sakarovitch C, Song F, et al. Evaluating non-randomised intervention studies. Health technology assessment (Winchester, England). 2003;7(27):iii–173.

8. Downs SH, Black N. The feasibility of creating a checklist for the assessment of the methodological quality both of randomised and non-randomised studies of health care interventions. J Epidemiol Community Health. 1998 Jun;52(6):377–84.

For peer review only

For peer review only - http://bmjopen.bmj.com/site/about/guidelines.xhtml

9. Malterud K. Systematic text condensation: a strategy for qualitative analysis. Scand J Public Health. 2012 Dec;40(8):795–805.

10. Dixon-Woods M, Cavers D, Agarwal S, Annandale E, Arthur A, Harvey J, et al. Conducting a critical interpretive synthesis of the literature on access to healthcare by vulnerable groups. BMC Medical Research Methodology. 2006 Jul 26;6(1):35.

11. Entwistle V, Firnigl D, Ryan M, Francis J, Kinghorn P. Which experiences of health care delivery matter to service users and why? A critical interpretive synthesis and conceptual map. J Health Serv Res Policy. 2012 Apr;17(2):70–8.

12. Schick-Makaroff K, MacDonald M, Plummer M, Burgess J, Neander W. What Synthesis Methodology Should I Use? A Review and Analysis of Approaches to Research Synthesis. AIMS Public Health. 2016;3(1):172–215.

13. Lewin S, Booth A, Glenton C, Munthe-Kaas H, Rashidian A, Wainwright M, et al. Applying GRADE-CERQual to qualitative evidence synthesis findings: introduction to the series. Implementation Science. 2018 Jan 25;13(1):2.

For peer review only

Supplementary Material 1

PRISMA-P Checklist

PRISMA-P (Preferred Reporting Items for Systematic review and Meta-Analysis Protocols) 2015 checklist

Section and topic	Item No	Checklist item	Line numbers
**ADMINISTRATIVE INFORMATION**			
Title:			
Identification	1a	Identify the report as a protocol of a systematic review	1-3
Update	1b	If the protocol is for an update of a previous systematic review, identify as such	n/a
Registration	2	If registered, provide the name of the registry (such as PROSPERO) and registration number	n/a
Authors:			
Contact	3a	Provide name, institutional affiliation, e-mail address of all protocol authors; provide physical mailing address of corresponding author	53-69
Contributions	3b	Describe contributions of protocol authors and identify the guarantor of the review	285-294
Amendments	4	If the protocol represents an amendment of a previously completed or published protocol, identify as such and list changes	n/a
Support:			
Sources	5a	Indicate sources of financial or other support for the review	296-298
Sponsor	5b	Provide name for the review funder and/or sponsor	296-298
Role of sponsor or funder	5c	Describe roles of funder(s), sponsor(s), and/or institution(s), if any, in developing the protocol	296-298
**INTRODUCTION**			
Rationale	6	Describe the rationale for the review in the context of what is already known	116-151
Objectives	7	Provide an explicit statement of the question(s) the review will address with reference to participants, interventions, comparators, and outcomes (PICO)	152-154
**METHODS**			
Eligibility criteria	8	Specify the study characteristics (such as PICO, study design, setting, time frame) and report characteristics (such as years considered, language, publication status) to be used as criteria for eligibility for the review	171-180

For peer review only

For peer review only - http://bmjopen.bmj.com/site/about/guidelines.xhtml

Information sources	9	Describe all intended information sources (such as electronic databases, contact with study authors, trial registers or other grey literature sources) with planned dates of coverage	182-187
Search strategy	10	Present draft of search strategy to be used for at least one electronic database, including planned limits, such that it could be repeated	Supplementary material 1.pdf
Study records:			
Data management	11a	Describe the mechanism(s) that will be used to manage records and data throughout the review	193-195
Selection process	11b	State the process that will be used for selecting studies (such as two independent reviewers) through each phase of the review (that is, screening, eligibility and inclusion in meta-analysis)	205-206
Data collection process	11c	Describe planned method of extracting data from reports (such as piloting forms, done independently, in duplicate), any processes for obtaining and confirming data from investigators	208-212
Data items	12	List and define all variables for which data will be sought (such as PICO items, funding sources), any pre-planned data assumptions and simplifications	214-229
Outcomes and prioritization	13	List and define all outcomes for which data will be sought, including prioritization of main and additional outcomes, with rationale	233-239
Risk of bias in individual studies	14	Describe anticipated methods for assessing risk of bias of individual studies, including whether this will be done at the outcome or study level, or both; state how this information will be used in data synthesis	241-255
Data synthesis	15a	Describe criteria under which study data will be quantitatively synthesised	257-262
	15b	If data are appropriate for quantitative synthesis, describe planned summary measures, methods of handling data and methods of combining data from studies, including any planned exploration of consistency (such as I^2, Kendall's τ)	260-262
	15c	Describe any proposed additional analyses (such as sensitivity or subgroup analyses, meta-regression)	260-261
	15d	If quantitative synthesis is not appropriate, describe the type of summary planned	257-259
Meta-bias(es)	16	Specify any planned assessment of meta-bias(es) (such as publication bias across studies, selective reporting within studies)	263-264
Confidence in cumulative evidence	17	Describe how the strength of the body of evidence will be assessed (such as GRADE)	266-278

It is strongly recommended that this checklist be read in conjunction with the PRISMA-P Explanation and Elaboration for important clarification on the items. Amendments to a review protocol should be tracked and dated. The copyright for PRISMA-P (including checklist) is held by the PRISMA-P Group and is distributed under a Creative Commons Attribution Licence 4.0.

From: Shamseer L, Moher D, Clarke M, Ghersi D, Liberati A, Petticrew M, Shekelle P, Stewart L, PRISMA-P Group. Preferred reporting items for systematic review and meta-analysis protocols (PRISMA-P) 2015: elaboration and explanation. BMJ. 2015 Jan 2;349(jan02 1):g7647.

For peer review only
For peer review only - http://bmjopen.bmj.com/site/about/guidelines.xhtml

Supplementary Material 2

Search Strategies

Medline via Ovid

Ovid MEDLINE(R) and Epub Ahead of Print, In-Process, In-Data-Review & Other Non-Indexed Citations, Daily and Versions <1946 to September 20, 2023>		
1	((clinical or medical or doctor) adj2 (student* or trainee* or school*)).ti,ab.	90738
2	exp Schools, Medical/ or exp Students, Medical/ or exp education, medical, undergraduate/	80136
3	1 or 2	126117
4	(Absen* or non-attendance or nonattendance or attend*).ti,ab.	1079900
5	(Placemen* or Lectur* or Semina* or online or virtual or teaching or "in person" or inperson or "face to face" or traditional or class*).ti,ab.	2761182
6	exp Teaching/	93309
7	5 or 6	2811415
8	(Exam* or Perform* or Attainment* or Result*).ti,ab.	15523220
9	exp academic performance/ or exp educational status/ or exp academic success/	65300
10	8 or 9	15540736
11	3 and 4 and 7 and 10	3160

Embase via Ovid

Embase <1974 to 2023 September 20>		
1	((clinical or medical or doctor) adj2 (student* or trainee* or school*)).ti,ab. or exp *medical school/ or exp *medical student/	131819
2	(Absen* or non-attendance or nonattendance or attend*).ti,ab. or exp *school attendance/	1459034
3	(Placemen* or Lectur* or Semina* or online or virtual or teaching or "in person" or inperson or "face to face" or traditional or class*).ti,ab. or exp *Teaching/	3657834
4	exp academic achievement/ or exp *educational status/ or (Exam* or Perform* or Attainment* or Result*).ti,ab.	20509010
5	1 and 2 and 3 and 4	5027
6	limit 5 to embase	1697

Web of Science

Web of Science Search Strategy (v0.1)<br># Database: Web of Science Core Collection<br># Entitlements:<br>- WOS.IC: 1993 to 2023<br>- WOS.CCR: 1985 to 2023<br>- WOS.SCI: 1900 to 2023<br>- WOS.AHCI: 1975 to 2023<br>- WOS.BHCI: 2008 to 2023<br>- WOS.BSCI: 2008 to 2023

For peer review only

- WOS.ESCI: 2018 to 2023<br>- WOS.ISTP: 1990 to 2023<br>- WOS.SSCI: 1956 to 2023<br>- WOS.ISSHP: 1990 to 2023<br>Date Run: Thu Sep 20, 2023		
1	TS=(Absen* OR "non-attendance" OR attendance)	1135903
2	TS= ((clinical OR medical OR doctor) near/2 (student* OR trainee* OR school*))	97987
3	TS=(Placemen* OR Lectur* OR Semina* OR online OR virtual OR teaching or "in person" or inperson or "face to face" or traditional or class*)	6854033
4	TS=(Exam* OR Perform* OR Attainment* OR Result* OR "academic performance" OR "educational status")	6854033
5	TS=(Exam* OR Perform* OR Attainment* OR Result* OR "academic performance" OR "educational status")	29046391
6	#4 AND #3 AND #2 AND #1	811

6

7 Scopus

Scopus<br>Date Run: Thu Sep 20, 2023	
1	TITLE-ABS-KEY (((absen* OR "non-attend*" OR attend* OR nonattend*) AND ((clinical OR medical OR doctor) W/2 (student* OR trainee* OR school*)) AND (placemen* OR lectur* OR semina* OR online OR virtual OR teaching OR "in person" OR inperson OR "face to face" OR traditional OR class*) AND (exam* OR perform* OR attainment* OR result* OR "academic performance" OR "educational status")))

8

9 British Education Index via EBSCOhost

Interface - EBSCOhost Research Databases<br>Search Screen - Advanced<br>Search Database - British Education Index<br><br>Expanders - Apply equivalent subjects<br>Search modes - Boolean/Phrase<br>Date Run: Thu Sep 20, 2023		
S1	TI ((clinical or medical or doctor) w2 (student* or trainee* or school*)) OR AB ((clinical or medical or doctor) w2 (student* or trainee* or school*))	4,076
S2	((DE "MEDICAL schools") OR (DE "MEDICAL students") OR (DE "MEDICAL education"))	9,405
S3	S1 OR S2	10,242
S4	TI ((Absen* or non-attendance or nonattendance or attend*)) OR ((Absen* or non-attendance or nonattendance or attend*))	7,649

For peer review only - http://bmjopen.bmj.com/site/about/guidelines.xhtml

S5	DE "SCHOOL attendance"	610
S6	S4 OR S5	7,649
S7	TI ((Placemen* or Lectur* or Semina* or online or virtual or teaching or "in person" or inperson or "face to face" or traditional or class*)) OR AB ((Placemen* or Lectur* or Semina* or online or virtual or teaching or "in person" or inperson or "face to face" or traditional or class*))	76,866
S8	DE "TEACHING"	6,709
S9	S7 OR S8	79,470
S10	TI ((Exam* or Perform* or Attainment* or Result*)) OR AB ((Exam* or Perform* or Attainment* or Result*))	80,327
S11	(DE "ACADEMIC achievement") OR (DE "EDUCATIONAL attainment")	10,645
S12	S10 OR S11	85,240
S13	S3 AND S6 AND S9 AND S12	101

For peer review only

ERIC via EBSCOhost

Interface - EBSCOhost Research Databases
Search Screen - Advanced
Search Database - ERIC

Expanders - Apply equivalent subjects
Search modes - Boolean/Phrase
Date Run: Thu Sep 20, 2023

S1	(AB (clinical or medical or doctor) w2 (student* or trainee* or school*)) OR (TI (clinical or medical or doctor) w2 (student* or trainee* or school*))	7,784
S2	TI (DE "Medical Schools") OR (DE "Medical Students") OR (DE "Medical Education")	397
S3	S1 OR S2	7,842
S4	(AB (Absen* or non-attendance or nonattendance or attend*)) OR (TI (Absen* or non-attendance or nonattendance or attend*))	73,324
S5	(AB (Placemen* or Lectur* or Semina* or online or virtual or teaching or "in person" or inperson or "face to face" or traditional or class*)) OR (TI (Placemen* or Lectur*	672,070

For peer review only - http://bmjopen.bmj.com/site/about/guidelines.xhtml

	or Semina* or online or virtual or teaching or "in person" or inperson or "face to face" or traditional or class*))	
S6	DE "Teaching Methods"	211,764
S7	S5 OR S6	737,112
S8	(AB (Exam* or Perform* or Attainment* or Result*)) OR (TI (Exam* or Perform* or Attainment* or Result*))	845,630
S9	DE "Academic Achievement"	94,882
S10	S8 OR S9	876,868
S11	S3 AND S4 AND S7 AND S10	138

13

For peer review only

For peer review only - http://bmjopen.bmj.com/site/about/guidelines.xhtml

BMJ Open

Changes in medical student attendance and its impact on student educational outcomes – A systematic review protocol

Journal:	*BMJ Open*
Manuscript ID	bmjopen-2023-078252.R3
Article Type:	Protocol
Date Submitted by the Author:	04-Jan-2024
Complete List of Authors:	Nagappan, Palaniappan Ganesh; University of Cambridge, School of Clinical Medicine<br>Brown, Samuel; University of Cambridge<br>McManus, Alex; University of Cambridge<br>Sayers, Sarah; University of Cambridge,<br>Absar, Shazia; University of Cambridge<br>Tan, Sapphire Rou Xi; University of Cambridge<br>Kuhn, Isla; University of Cambridge<br>Tulinius, Charlotte; University of Cambridge<br>Lau, Edward; University of Cambridge
**Primary Subject Heading**:	Medical education and training
Secondary Subject Heading:	Communication, Ethics, Evidence based practice, Health policy
Keywords:	EDUCATION & TRAINING (see Medical Education & Training), Health Education, MEDICAL EDUCATION & TRAINING

SCHOLARONE™
Manuscripts

Changes in medical student attendance and its impact on student educational outcomes – A systematic review protocol

For peer review only

Contents

Administrative information 3
- Title 3
- Authors 3

Abstract 3
- Introduction 3
- Methods and analysis 4
- Ethics and dissemination 4

Article Summary 4
- Strengths and limitations of this study 4
- Keywords 4

Introduction 4
- Rationale 4

Research Question 5
- Objectives 5

Methods 6
- Eligibility criteria 6
- Information sources 6
- Search strategy 6
- Study records 6
  - Data management 6
  - Selection process 6

Data collection process 7
Data items 7
Outcomes and prioritization 7
Risk of bias in individual studies 8
Data synthesis 8
Meta-bias(es) 8
Confidence in cumulative evidence 8
Ethics and dissemination 9
Patient and Public Involvement 9
Author Contributions 9
Funding 9
Competing interests' statement 9
References 10
Supplementary material 11
Search Strategies 11
Medline via Ovid 11
Embase via Ovid 11
Web of Science 11
Scopus 12
British Education Index via EBSCOhost 12
ERIC via EBSCOhost 13

For peer review only - http://bmjopen.bmj.com/site/about/guidelines.xhtml

Administrative information

Title

Changes in medical student attendance and its impact on student educational outcomes – A systematic review protocol

Authors

Dr Palaniappan Ganesh Nagappan. University of Cambridge: pgn25@cam.ac.uk

Mr Samuel Brown. University of Cambridge: sb2382@cam.ac.uk

Mr Alex McManus. University of Cambridge: am2776@cam.ac.uk

Miss Sarah Sayers. University of Cambridge: sbs51@cam.ac.uk

Miss Shazia Absar. University of Cambridge: sha36@cam.ac.uk

Dr Sapphire Tan Rou Xi, University of Cambridge: rxst2@cam.ac.uk

Miss Isla Kuhn. University of Cambridge: ilk21@cam.ac.uk

Dr Edward Lau. University of Cambridge: ejsl2@cam.ac.uk

Dr Charlotte Tulinius. University of Cambridge: ct419@medschl.cam.ac.uk

Corresponding author:

Dr Palaniappan Ganesh Nagappan
Emmanuel College
Cambridge, CB23AP, UK
+447472659583
Pgn25@cam.ac.uk

Word Count: 1921 words

Abstract

Introduction

The COVID-19 pandemic has had a significant impact on medical education, with many institutions shifting to online learning to ensure the safety of students and staff. However, there has been a decline in in-person attendance at medical schools across the UK and worldwide following the relaxation of social distancing rules and the reinstation of in-person teaching. Importantly, this trend has been observed prior to the pandemic. Whilst reflected within the literature, there is currently no systematic review describing these changes. We aim to find out how medical students' attendance is changing as documented within the literature and its impact on their educational outcomes.

For peer review only

Methods and analysis

This systematic review will follow the guidelines of the Centre of Research and Dissemination, Moose, and PRISMA. We will search the major databases of Medline via Ovid, Embase via Ovid, Scopus, Web of Science, British Education Index via EBSCOhost and ERIC via EBSCOhost.

Two reviewers will independently screen each paper and extract data, with a third reviewer for dispute resolution. All studies reporting on medical students from various universities, both graduate and undergraduate and describing changes in attendance and/or students' educational outcomes will be included. Risk of bias in individual studies will be assessed using the Newcastle-Ottawa Scale and confidence in cumulative evidence will be evaluated using the GRADE-CERQual approach. A narrative synthesis of the findings from all included studies will be reported.

Ethics and dissemination

Ethical approval is not required for this systematic review of existing publicly available literature. We will subsequently aim to publish the results of this systematic review in a peer-reviewed journal.

Article Summary

Strengths and limitations of this study

- The study's adherence to comprehensive methodology, following established guidelines like the Centre of Research and Dissemination, Moose, and PRISMA, ensures a systematic and rigorous approach, enhancing credibility and reliability.
- The inclusion of diverse research types (qualitative, quantitative, mixed methods) provides a comprehensive understanding, allowing triangulation of evidence and strengthening the validity of conclusions.
- Independent data management and extraction by two pairs of reviewers reduce biases and enhance objectivity, leading to more robust and credible research outcomes.
- Being a retrospective, observational research design, systematic reviews are subject to systematic and random error. We hope to minimise this by strict adherence to the guidelines mentioned above.
- Potential language bias could lead to oversight of relevant studies, and addressing these limitations is essential for a more comprehensive analysis.

Keywords

- Medical Students (MeSH Term: Students, Medical)
- Attendance (MeSH Term: Absenteeism)
- Performance (MeSH Term: Academic Performance)

For peer review only - http://bmjopen.bmj.com/site/about/guidelines.xhtml

Introduction

Rationale

Post-pandemic, following a shift back to in-person teaching, the level of medical student attendance at many higher education institutions, including medical schools across the UK seems to have fallen dramatically. Teachers and leads across all subjects report a very low attendance rate at small group and larger group teaching and whole cohort lectures and a similar phenomenon has been reported across other medical schools in the UK. Although the literature is still limited on this development, this appears to be a worldwide observation (1). At our institution and within the literature, this change in the attendance was noted to occur prior to the pandemic (2), but became significantly more noticeable following the return to in-person teaching post-pandemic.

Before the COVID-19 pandemic, most students would have expected to be on clinical placements all day, but the pandemic introduced the need for replacement of in-person education with creative experiments involving learning online and from home, and the application of telemedicine (3). This need acted as a catalyst and accelerated the development of online learning. With the shift back to in-person teaching, some students are now actively opting out of in-person teaching (4). Whilst we acknowledge that the COVID-19 pandemic may have been a factor contributing to this observation, as this was noted prior to the return to in-person teaching, there may be other explanations and the changes in attendance may reflect a shift in the direction of medical education, raising the question of whether attendance is a good measure of high quality medical education (5). For example, this could be explained by students increasingly realising that attending core curriculum teaching in person is no longer an absolute necessity for successful completion of the curriculum (1) or an increase in the utility and availability of online learning materials. A greater understanding of what these changes in attendance are, along with why the changes in attendance are happening and its impact on student educational outcomes would be valuable in determining whether further action is required to maintain high standards of medical education.

Identifying areas where institutions can help support this evolving learning process is essential to continue to provide a holistic education. Without this, students' learning may be at risk of becoming overtly focused in some areas and less in others. Higher education online learning was available before the COVID-19 pandemic but this was often variable and delivered asynchronously (6). Exploring previously adopted teaching strategies and their impact on student educational outcomes can help to guide the development of innovative models of teaching to ensure continuous high-quality education.

To date, there are no systematic reviews published that describe the changes in attendance documented within the literature, and its subsequent influence on student education outcomes. This systematic review aims to explore and explain the ongoing changes in medical student attendance that have been noted to start prior to but appear to be exacerbated by the pandemic and describe the potential impacts this may have on student educational outcomes.

Research Question

Research question: in what ways is student attendance to in-person teaching sessions changing and what are the impacts of this on student educational outcomes?

For peer review only - http://bmjopen.bmj.com/site/about/guidelines.xhtml

Objectives

Primary aim

- To describe the changes in medical student attendance at in-person teaching sessions

An initial scope of the literature revealed that changes in medical school attendance began prior to the COVID-19 pandemic. As a result, we have decided to remove the secondary aim 'to describe the impact of the COVID-19 pandemic on medical student attendance for in-person teaching sessions' to ensure that we captured all changes in student attendance, not just those following the pandemic. This leads to the following secondary aims for this literature review.

Secondary aims

- To explain the changes to medical student attendance at in-person teaching sessions.
- To describe the impact of the changes in attendance on medical student educational outcomes.
- To describe strategies adopted by educators in light of these changes to medical student attendance at in-person teaching sessions.

Methods

The design and methods used for this systematic review comply with Centre of Research and Dissemination Guidelines, Meta-analyses of Observational Studies in Epidemiology (Moose) and are reported in line with Preferred Reporting Items for Systematic Reviews and Meta-Analyses (PRISMA). Eligibility criteria were informed using the SPIDER and MOOSE guidelines. This protocol adheres to the PRISMA-P guidelines ("Supplementary Material 1.pdf").

Eligibility criteria

(S) Sample: Medical students in both graduate and undergraduate medical curriculums across all types of universities (private and public)

(PI) The phenomenon of Interest: Changes in attendance, educational outcomes, or both.

(E) Evaluation: 1. Recorded or anecdotal evidence of changes in attendance and 2. Comparison between 2 or more sets of examination scores or any other performance-based measures (including interest, satisfaction and confidence rates).

(D) Design: Primary studies excluding grey literature.

(R) Research type: Primary studies of qualitative, quantitative and mixed-methods research could be searched for, not including systematic reviews, literature reviews or metanalysis.

For peer review only - http://bmjopen.bmj.com/site/about/guidelines.xhtml

Information sources

The search will employ topic-based strategies designed for each database from inception to 20th September 2023. There will be no language or geographical restrictions.

Databases:

EMBASE via OVID, MEDLINE via OVID, Scopus, Web of Science Core Collection, British Education Index via EBSCOhost, ERIC via EBSCOhost

Search strategy

The detailed search strategies for the respective databases can be found under Supplementary Materials (“Supplementary Material 2.pdf”).

Study records

Data management

Records will be managed through Endnote and NVIVO.

Selection process

Inclusion criteria:

- Medical students studying in medical schools accredited by their country’s governing body.
- Language of Publication: all languages
- Settings: Hospitals, medical schools
- At least one of the following outcomes: 1. attendance rates, 2. performance measures, and 3. solutions.

Exclusion criteria:

- Grey literature
- Secondary research

The screening will be performed independently by two pairs of reviewers (SS/SA; SB/AM, with each pair covering 50% of the papers, with a third individual to resolve any disagreements.

Data collection process

Using a standardised form, two pairs of reviewers (SS/SA; SB/AM) will extract the data independently, with each pair covering 50% of the papers. A third reviewer (PN) will independently check the data for consistency and clarity.

For peer review only - http://bmjopen.bmj.com/site/about/guidelines.xhtml

Data items

The data extracted will include the following summary data:

- Sample characteristics
- Sample size
- Study date
- Medium of lecture / programme / teaching
- Stage of medical education training
- Academic outcome / performance scores
- Academic outcome / performance measure used
- Confidence, interest and satisfaction rates
- Attendance rates (stratified by in-person and otherwise)
- Access rates to online materials
- Solutions / interventions used
- Association between attendance rates and academic outcomes / performance
- Students' perception
- Other outcomes

This will be in addition to the critical arguments raised during the process. Both qualitative and quantitative data will be collected for narrative synthesis.

Outcomes and prioritization

1. Recorded or anecdotal evidence of changes in attendance at live learning opportunities both in person and otherwise. This would include formative and summative assessments as well if it is used as a "technique" for learning rather than only as a measurement of performance. An example of an in-person teaching assessment is an OSCE setup.

2. Comparison of examination scores, performance-based measures, or educational outcomes with the implementation of a learning technique or intervention.

Risk of bias in individual studies

The risk of bias for each included trial will be independently assessed by the same initial reviewers. The fifth reviewer will mediate in situations of disagreement. Cohen's κ will be used to assess agreement between reviewers. All tools and processes will be piloted before use. The risk of bias will be assessed using the Newcastle-Ottawa Scale (7,8).

The following assessments will then be made, depending on the study type:

Qualitative studies: The reviewers will assess the appropriateness of data sources and analytical processes, the study's transparency, consideration of context/setting, and the study's consideration of researchers' reflexivity and positioning.

For peer review only - http://bmjopen.bmj.com/site/about/guidelines.xhtml

Descriptive cross-sectional quantitative studies: The reviewers will assess the appropriateness of the study's sampling strategy and the representativeness of the sample, the use of appropriate measurement instruments, and the acceptability of the response rate.

Nonrandomised quantitative studies: The reviewers will assess the study's minimisation of selection bias, use of appropriate measurement instruments, use of comparable groups across study conditions, and the completeness of outcome data.

Data synthesis

Qualitative Data: Qualitative data will be imported into VIVO software. Malterud's systematic text condensation as thematic analysis will be conducted by two reviewers independently with a discussion following this. (9–12)

Quantitative Data: The data collected will undergo narrative synthesis. A meta-analysis will be considered should the quantitative data be sufficiently homogenous.

Meta-bias(es)

N/A

Confidence in cumulative evidence

Confidence in discrete review findings will be assessed using the recently developed Grading of Recommendations, Assessment, Development and Evaluation - Confidence in the Evidence from Reviews of Qualitative Research (GRADE-CERQual) (13). Assessment of confidence in a given review finding involves evaluating how likely it is that the finding represents a real phenomenon, i.e. factors leading to changes in attendance, performance and their relationship with each other. This assessment will be based upon an evaluation of the following: (i) methodological limitations of the primary studies contributing to the finding, (ii) the relevance of the primary contributing studies regarding the objectives of the systematic review, (iii) the coherence of the finding, and (iv) the adequacy of data supporting the finding.

A summary table will list each review finding—primary contributing studies, evaluations of the above four domains, an overall confidence rating (high, moderate, low, or very low), and an explanation of the rating judgement.

Ethics and dissemination

Ethical approval is not required for this systematic review of existing publicly available literature. We will subsequently aim to publish the results of this systematic review in a peer-reviewed journal.

Patient and Public Involvement

None.

For peer review only - http://bmjopen.bmj.com/site/about/guidelines.xhtml

Estimated timeline

The estimated timeline of the systematic review is as described in Table 1.

Stage	Planned period
Title and abstract screening	December 2023
Full text screening	December 2023
Data extraction and quality assessment	January 2024
Statistical analysis	February 2024
Manuscript writing, revision and submission	March 2024

Table 1: Estimated timeline

For peer review only

Author Contributions

PGN: Drafted the proposal and conceptualisation of the topic.

SB: Data collection and refining of the topic.

AM: Data collection and refining of the topic.

SS: Data collection and refining of the topic.

SA: Data collection and refining of the topic.

ST: Conceptualisation of the topic.

IK: Reviewing and critiquing to improve research methodology with the drafting of the search strategy.

CT: Conceptualisation of topic and supervision.

EL: Conceptualisation of topic and supervision.

Funding

This research received no specific grant from any funding agency in the public, commercial or not-for-profit sectors. However, the necessary NVivo licenses will be available from the University of Cambridge.

Competing interests' statement

There are no known competing interests.

References

1. Gardner G, Feldman M, Santen SA, Mui P, Biskobing D. Determinants and Outcomes of In-person Lecture Attendance in Medical School. Med Sci Educ. 2022 Aug;32(4):883–90.

2. Emahiser J, Nguyen J, Vanier C, Sadik A. Study of Live Lecture Attendance, Student Perceptions and Expectations. Med Sci Educ. 2021 Apr;31(2):697–707.

For peer review only - http://bmjopen.bmj.com/site/about/guidelines.xhtml

3. Park H, Shim S, Lee YM. A scoping review on adaptations of clinical education for medical students during COVID-19. Prim Care Diabetes. 2021 Dec;15(6):958–76.

4. Jhajj S, Kaur P, Jhajj P, Ramadan A, Jain P, Upadhyay S, et al. Impact of Covid-19 on Medical Students around the Globe. J Community Hosp Intern Med Perspect. 2022;12(4):1–6.

5. Kanter SL. To Be There or Not To Be There: Is Attendance Really the Question? Academic Medicine. 2012 Jun;87(6):679.

6. Tallent-Runnels MK, Thomas JA, Lan WY, Cooper S, Ahern TC, Shaw SM, et al. Teaching Courses Online: A Review of the Research. Review of Educational Research. 2006 Mar 1;76(1):93–135.

7. Deeks JJ, Dinnes J, D'Amico R, Sowden AJ, Sakarovitch C, Song F, et al. Evaluating non-randomised intervention studies. Health technology assessment (Winchester, England). 2003;7(27):iii–173.

8. Downs SH, Black N. The feasibility of creating a checklist for the assessment of the methodological quality both of randomised and non-randomised studies of health care interventions. J Epidemiol Community Health. 1998 Jun;52(6):377–84.

9. Malterud K. Systematic text condensation: a strategy for qualitative analysis. Scand J Public Health. 2012 Dec;40(8):795–805.

10. Dixon-Woods M, Cavers D, Agarwal S, Annandale E, Arthur A, Harvey J, et al. Conducting a critical interpretive synthesis of the literature on access to healthcare by vulnerable groups. BMC Medical Research Methodology. 2006 Jul 26;6(1):35.

11. Entwistle V, Firnigl D, Ryan M, Francis J, Kinghorn P. Which experiences of health care delivery matter to service users and why? A critical interpretive synthesis and conceptual map. J Health Serv Res Policy. 2012 Apr;17(2):70–8.

12. Schick-Makaroff K, MacDonald M, Plummer M, Burgess J, Neander W. What Synthesis Methodology Should I Use? A Review and Analysis of Approaches to Research Synthesis. AIMS Public Health. 2016;3(1):172–215.

13. Lewin S, Booth A, Glenton C, Munthe-Kaas H, Rashidian A, Wainwright M, et al. Applying GRADE-CERQual to qualitative evidence synthesis findings: introduction to the series. Implementation Science. 2018 Jan 25;13(1):2.

For peer review only

Supplementary Material 1

PRISMA-P Checklist

PRISMA-P (Preferred Reporting Items for Systematic review and Meta-Analysis Protocols) 2015 checklist

Section and topic	Item No	Checklist item	Line numbers
**ADMINISTRATIVE INFORMATION**			
Title:			
Identification	1a	Identify the report as a protocol of a systematic review	1-3
Update	1b	If the protocol is for an update of a previous systematic review, identify as such	n/a
Registration	2	If registered, provide the name of the registry (such as PROSPERO) and registration number	n/a
Authors:			
Contact	3a	Provide name, institutional affiliation, e-mail address of all protocol authors; provide physical mailing address of corresponding author	53-69
Contributions	3b	Describe contributions of protocol authors and identify the guarantor of the review	287-296
Amendments	4	If the protocol represents an amendment of a previously completed or published protocol, identify as such and list changes	n/a
Support:			
Sources	5a	Indicate sources of financial or other support for the review	298-300
Sponsor	5b	Provide name for the review funder and/or sponsor	298-300
Role of sponsor or funder	5c	Describe roles of funder(s), sponsor(s), and/or institution(s), if any, in developing the protocol	298-300
**INTRODUCTION**			
Rationale	6	Describe the rationale for the review in the context of what is already known	116-151
Objectives	7	Provide an explicit statement of the question(s) the review will address with reference to participants, interventions, comparators, and outcomes (PICO)	152-154
**METHODS**			
Eligibility criteria	8	Specify the study characteristics (such as PICO, study design, setting, time frame) and report characteristics (such as years considered, language, publication status) to be used as criteria for eligibility for the review	171-180

For peer review only

For peer review only - http://bmjopen.bmj.com/site/about/guidelines.xhtml

Information sources	9	Describe all intended information sources (such as electronic databases, contact with study authors, trial registers or other grey literature sources) with planned dates of coverage	182-187
Search strategy	10	Present draft of search strategy to be used for at least one electronic database, including planned limits, such that it could be repeated	Supplementary material 1.pdf
Study records:			
Data management	11a	Describe the mechanism(s) that will be used to manage records and data throughout the review	193-195
Selection process	11b	State the process that will be used for selecting studies (such as two independent reviewers) through each phase of the review (that is, screening, eligibility and inclusion in meta-analysis)	205-206
Data collection process	11c	Describe planned method of extracting data from reports (such as piloting forms, done independently, in duplicate), any processes for obtaining and confirming data from investigators	208-212
Data items	12	List and define all variables for which data will be sought (such as PICO items, funding sources), any pre-planned data assumptions and simplifications	214-229
Outcomes and prioritization	13	List and define all outcomes for which data will be sought, including prioritization of main and additional outcomes, with rationale	233-239
Risk of bias in individual studies	14	Describe anticipated methods for assessing risk of bias of individual studies, including whether this will be done at the outcome or study level, or both; state how this information will be used in data synthesis	241-255
Data synthesis	15a	Describe criteria under which study data will be quantitatively synthesised	257-262
	15b	If data are appropriate for quantitative synthesis, describe planned summary measures, methods of handling data and methods of combining data from studies, including any planned exploration of consistency (such as I^2, Kendall's τ)	260-262
	15c	Describe any proposed additional analyses (such as sensitivity or subgroup analyses, meta-regression)	260-261
	15d	If quantitative synthesis is not appropriate, describe the type of summary planned	257-259
Meta-bias(es)	16	Specify any planned assessment of meta-bias(es) (such as publication bias across studies, selective reporting within studies)	263-264
Confidence in cumulative evidence	17	Describe how the strength of the body of evidence will be assessed (such as GRADE)	266-278

It is strongly recommended that this checklist be read in conjunction with the PRISMA-P Explanation and Elaboration for important clarification on the items. Amendments to a review protocol should be tracked and dated. **The copyright for PRISMA-P (including checklist) is held by the PRISMA-P Group and is distributed under a Creative Commons Attribution Licence 4.0.**

From: Shamseer L, Moher D, Clarke M, Ghersi D, Liberati A, Petticrew M, Shekelle P, Stewart L, PRISMA-P Group. Preferred reporting items for systematic review and meta-analysis protocols (PRISMA-P) 2015: elaboration and explanation. BMJ. 2015 Jan 2;349(jan02 1):g7647.

For peer review only

For peer review only - http://bmjopen.bmj.com/site/about/guidelines.xhtml

Supplementary Material 2

Search Strategies

Medline via Ovid

Ovid MEDLINE(R) and Epub Ahead of Print, In-Process, In-Data-Review & Other Non-Indexed Citations, Daily and Versions <1946 to September 20, 2023>		
1	((clinical or medical or doctor) adj2 (student* or trainee* or school*)).ti,ab.	90738
2	exp Schools, Medical/ or exp Students, Medical/ or exp education, medical, undergraduate/	80136
3	1 or 2	126117
4	(Absen* or non-attendance or nonattendance or attend*).ti,ab.	1079900
5	(Placemen* or Lectur* or Semina* or online or virtual or teaching or "in person" or inperson or "face to face" or traditional or class*).ti,ab.	2761182
6	exp Teaching/	93309
7	5 or 6	2811415
8	(Exam* or Perform* or Attainment* or Result*).ti,ab.	15523220
9	exp academic performance/ or exp educational status/ or exp academic success/	65300
10	8 or 9	15540736
11	3 and 4 and 7 and 10	3160

Embase via Ovid

Embase <1974 to 2023 September 20>		
1	((clinical or medical or doctor) adj2 (student* or trainee* or school*)).ti,ab. or exp *medical school/ or exp *medical student/	131819
2	(Absen* or non-attendance or nonattendance or attend*).ti,ab. or exp *school attendance/	1459034
3	(Placemen* or Lectur* or Semina* or online or virtual or teaching or "in person" or inperson or "face to face" or traditional or class*).ti,ab. or exp *Teaching/	3657834
4	exp academic achievement/ or exp *educational status/ or (Exam* or Perform* or Attainment* or Result*).ti,ab.	20509010
5	1 and 2 and 3 and 4	5027
6	limit 5 to embase	1697

Web of Science

Web of Science Search Strategy (v0.1)
Database: Web of Science Core Collection
Entitlements:
- WOS.IC: 1993 to 2023
- WOS.CCR: 1985 to 2023
- WOS.SCI: 1900 to 2023
- WOS.AHCI: 1975 to 2023
- WOS.BHCI: 2008 to 2023
- WOS.BSCI: 2008 to 2023

For peer review only

- WOS.ESCI: 2018 to 2023<br>- WOS.ISTP: 1990 to 2023<br>- WOS.SSCI: 1956 to 2023<br>- WOS.ISSHP: 1990 to 2023<br>Date Run: Thu Sep 20, 2023		
1	TS=(Absen* OR "non-attendance" OR attendance)	1135903
2	TS= ((clinical OR medical OR doctor) near/2 (student* OR trainee* OR school*))	97987
3	TS=(Placemen* OR Lectur* OR Semina* OR online OR virtual OR teaching or "in person" or inperson or "face to face" or traditional or class*)	6854033
4	TS=(Exam* OR Perform* OR Attainment* OR Result* OR "academic performance" OR "educational status")	6854033
5	TS=(Exam* OR Perform* OR Attainment* OR Result* OR "academic performance" OR "educational status")	29046391
6	#4 AND #3 AND #2 AND #1	811

Scopus

Scopus<br>Date Run: Thu Sep 20, 2023	
1	TITLE-ABS-KEY (((absen* OR "non-attend*" OR attend* OR nonattend*) AND ((clinical OR medical OR doctor) W/2 (student* OR trainee* OR school*)) AND (placemen* OR lectur* OR semina* OR online OR virtual OR teaching OR "in person" OR inperson OR "face to face" OR traditional OR class*) AND (exam* OR perform* OR attainment* OR result* OR "academic performance" OR "educational status")))

British Education Index via EBSCOhost

Interface - EBSCOhost Research Databases<br>Search Screen - Advanced<br>Search Database - British Education Index<br><br>Expanders - Apply equivalent subjects<br>Search modes - Boolean/Phrase<br>Date Run: Thu Sep 20, 2023		
S1	TI ((clinical or medical or doctor) w2 (student* or trainee* or school*)) OR AB ((clinical or medical or doctor) w2 (student* or trainee* or school*))	4,076
S2	((DE "MEDICAL schools") OR (DE "MEDICAL students") OR (DE "MEDICAL education"))	9,405
S3	S1 OR S2	10,242
S4	TI ((Absen* or non-attendance or nonattendance or attend*)) OR ((Absen* or non-attendance or nonattendance or attend*))	7,649

For peer review only - http://bmjopen.bmj.com/site/about/guidelines.xhtml

S5	DE "SCHOOL attendance"	610
S6	S4 OR S5	7,649
S7	TI ((Placemen* or Lectur* or Semina* or online or virtual or teaching or "in person" or inperson or "face to face" or traditional or class*)) OR AB ((Placemen* or Lectur* or Semina* or online or virtual or teaching or "in person" or inperson or "face to face" or traditional or class*))	76,866
S8	DE "TEACHING"	6,709
S9	S7 OR S8	79,470
S10	TI ((Exam* or Perform* or Attainment* or Result*)) OR AB ((Exam* or Perform* or Attainment* or Result*))	80,327
S11	(DE "ACADEMIC achievement") OR (DE "EDUCATIONAL attainment")	10,645
S12	S10 OR S11	85,240
S13	S3 AND S6 AND S9 AND S12	101

ERIC via EBSCOhost

Interface - EBSCOhost Research Databases
Search Screen - Advanced
Search Database - ERIC

Expanders - Apply equivalent subjects
Search modes - Boolean/Phrase
Date Run: Thu Sep 20, 2023

S1	(AB (clinical or medical or doctor) w2 (student* or trainee* or school*)) OR (TI (clinical or medical or doctor) w2 (student* or trainee* or school*))	7,784
S2	TI (DE "Medical Schools") OR (DE "Medical Students") OR (DE "Medical Education")	397
S3	S1 OR S2	7,842
S4	(AB (Absen* or non-attendance or nonattendance or attend*)) OR (TI (Absen* or non-attendance or nonattendance or attend*))	73,324
S5	(AB (Placemen* or Lectur* or Semina* or online or virtual or teaching or "in person" or inperson or "face to face" or traditional or class*)) OR (TI (Placemen* or Lectur*	672,070

For peer review only - http://bmjopen.bmj.com/site/about/guidelines.xhtml

	or Semina* or online or virtual or teaching or "in person" or inperson or "face to face" or traditional or class*))	
S6	DE "Teaching Methods"	211,764
S7	S5 OR S6	737,112
S8	(AB (Exam* or Perform* or Attainment* or Result*)) OR (TI (Exam* or Perform* or Attainment* or Result*))	845,630
S9	DE "Academic Achievement"	94,882
S10	S8 OR S9	876,868
S11	S3 AND S4 AND S7 AND S10	138

13

For peer review only

For peer review only - http://bmjopen.bmj.com/site/about/guidelines.xhtml
